# Fast, flexible gene cluster family delineation with IGUA

**DOI:** 10.1093/nar/gkag975

**Published:** 2026-10-13

**Authors:** Martin Larralde, Josefin Blom, Hadrien Gourlé, Hale-Seda Radoykova, Lucas Paoli, Georg Zeller, Laura M Carroll

**Affiliations:** Molecular Systems Biology Unit, European Molecular Biology Laboratory (EMBL), Heidelberg, Baden-Württemberg 69117, Germany; Leiden University Center for Infectious Diseases (LUCID), Leiden University Medical Center, Leiden, South Holland 2333ZA, Netherlands; Department of Clinical Microbiology, Umeå University, Umeå, Västerbotten 90187, Sweden; Science for Life Laboratory (SciLifeLab), Umeå University, Umeå, Västerbotten 90187, Sweden; Laboratory for Molecular Infection Medicine Sweden (MIMS), Umeå University, Umeå, Västerbotten 90187, Sweden; Umeå Centre for Microbial Research (UCMR), Umeå University, Umeå, Västerbotten 90187, Sweden; Department of Clinical Microbiology, Umeå University, Umeå, Västerbotten 90187, Sweden; Science for Life Laboratory (SciLifeLab), Umeå University, Umeå, Västerbotten 90187, Sweden; Laboratory for Molecular Infection Medicine Sweden (MIMS), Umeå University, Umeå, Västerbotten 90187, Sweden; Umeå Centre for Microbial Research (UCMR), Umeå University, Umeå, Västerbotten 90187, Sweden; Global Health Institute, School of Life Science, École Polytechnique Fédérale de Lausanne (EPFL), Lausanne, Vaud 1015, Switzerland; Global Health Institute, School of Life Science, École Polytechnique Fédérale de Lausanne (EPFL), Lausanne, Vaud 1015, Switzerland; Molecular Systems Biology Unit, European Molecular Biology Laboratory (EMBL), Heidelberg, Baden-Württemberg 69117, Germany; Leiden University Center for Infectious Diseases (LUCID), Leiden University Medical Center, Leiden, South Holland 2333ZA, Netherlands; Center for Microbiome Analyses and Therapeutics (CMAT), Leiden University Medical Center, Leiden, South Holland 2333ZA, Netherlands; Department of Clinical Microbiology, Umeå University, Umeå, Västerbotten 90187, Sweden; Science for Life Laboratory (SciLifeLab), Umeå University, Umeå, Västerbotten 90187, Sweden; Laboratory for Molecular Infection Medicine Sweden (MIMS), Umeå University, Umeå, Västerbotten 90187, Sweden; Umeå Centre for Microbial Research (UCMR), Umeå University, Umeå, Västerbotten 90187, Sweden; Integrated Science Lab (IceLab), Umeå University, Umeå, Västerbotten 90187, Sweden

## Abstract

Prokaryotic genomes harbor a variety of functional elements encoded as contiguous multi-gene clusters. To identify homologous gene clusters, computational tools that account for multi-gene architectures are needed to group gene clusters into Gene Cluster Families (GCFs). However, existing GCF delineation methods do not scale well to large datasets and are often limited to certain subclasses of gene clusters. Here, we present IGUA (Iterative Gene clUster Analysis; https://github.com/zellerlab/IGUA), a scalable, flexible GCF delineation method for genomic segments with multi-gene architectures. On a biosynthetic gene cluster (BGC) clustering task, IGUA is 29× and 10× faster than the state-of-the-art (BiG-SCAPE v2 and BiG-SLiCE v2, respectively; 28 CPUs), without sacrificing accuracy. To highlight its scalability, we use IGUA to cluster >2.8 million BGCs from ≈1 million prokaryotic genomes in <18 h (*n* = 2 829 071 BGCs to 56 960 GCFs). To showcase its utility beyond BGC clustering, we use IGUA to cluster five additional prokaryotic gene cluster types (secretion systems, prophages, genomic islands, phage-plasmids, and carbohydrate active enzyme [CAZyme] gene clusters). Overall, IGUA represents a versatile GCF delineation tool with unmatched computational efficiency and flexibility, enabling (meta)genomic mining applications at unprecedented scales.

## Graphical Abstract

**Graphical Abstract:**
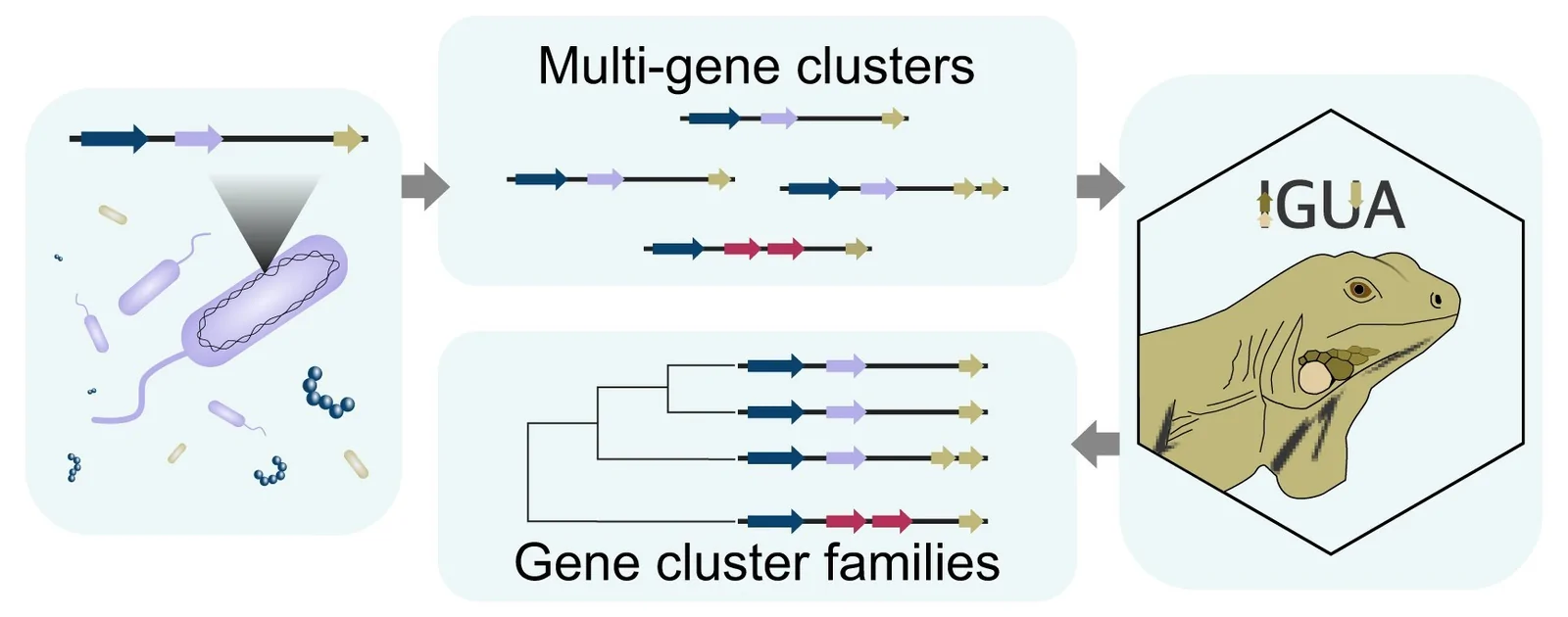

## Introduction

In prokaryotic genomes, genome organization is indicative of function, and proteins that can be functionally grouped into the same pathway tend to be encoded as genes that are in close proximity to each other in the genome [1, 2]. A variety of bioinformatic methods/tools have been developed for detecting multi-gene clusters in prokaryotic (meta)genomes [3–5], including specialist tools designed to detect specific types of prokaryotic gene clusters, such as genomic islands [6–9], secretion systems [10, 11], prophages [12–17], phage defense systems [18–20], carbohydrate active enzyme (CAZyme) gene clusters [21], and biosynthetic gene clusters (BGCs) [22–26]. When applied to the exponentially increasing amount of prokaryotic (meta)genomes available in public databases [27], these bioinformatic tools are generating larger gene cluster datasets and databases than ever before [28–31].

In order to study the evolution and diversity of prokaryotic gene clusters at the magnitude enabled by current databases, scalable bioinformatic methods for identifying and grouping homologous gene clusters are paramount [32–34]. These groupings of homologous gene clusters—referred to hereafter as Gene Cluster Families (GCFs)—can be used to e.g. filter out redundant gene clusters, assign traits or functions to unknown gene clusters, and identify putative novel gene clusters [32, 34]. Importantly, homologous gene clusters within a GCF can vary in terms of their genetic content and gene arrangement/structure (e.g. due to gene gain/loss and reshuffling) [32, 35–40]. As such, specialized GCF delineation tools are needed, as off-the-shelf sequence alignment/clustering methods, which do not account for accessory gene presence/absence and gene rearrangement within gene clusters (e.g. BLAST, MMseqs2, DIAMOND and CD-HIT) [41–44], are inadequate for GCF delineation.

The GCF concept has been popular within the BGC space for over a decade [26, 45], where GCFs are used to group BGCs encoding the same/similar secondary metabolites [32–34]. Consequently, to date, state-of-the-art bioinformatic methods for GCF delineation have been designed specifically for BGCs, with BiG-SCAPE [32] and BiG-SLiCE [33] being arguably the most established. BiG-SCAPE works by taking BGC nucleotide sequences (from antiSMASH, perhaps the most popular BGC detection tool for over a decade, and/or MIBiG, a database of experimentally validated BGCs) [22, 32, 46] as input, translating them into protein sequences, and representing each as a string of Pfam protein domains [32, 47]. BGCs are then clustered into GCFs using a combination of three distance metrics (Jaccard index, adjacency index, and domain sequence similarity index), weighted differently depending on the biosynthetic class of the BGCs [32]. While accurate and highly sensitive, BiG-SCAPE is slow, and its quadratic runtime complexity prevents it from scaling to large datasets (i.e. >10^4^ BGCs) [33]. To overcome this limitation, BiG-SLiCE was designed to delineate GCFs in a similar fashion, but with algorithmic changes that sacrifice some accuracy in favor of speed [33].

BiG-SCAPE and BiG-SLiCE have enabled GCF delineation to become a key component of many bioinformatic workflows for BGC discovery and functional prediction [34]. However, for other types of prokaryotic gene clusters, similar needs for grouping and redundancy removal exist, but the GCF concept has largely been unadopted, likely due to a lack of analogous GCF delineation methods. BiG-SCAPE and BiG-SLiCE themselves are BGC-specific and designed to work optimally with the output of antiSMASH and/or MIBiG [22, 32, 33, 46]; they cannot be used for GCF delineation tasks beyond BGCs, nor are they designed to be used with the output of alternative BGC detection tools (e.g. newer machine learning [ML]-based methods) [23, 24, 26]. Further, both tools are dependent on the presence of Pfam protein domains for clustering [32, 33, 47]. Gene clusters with low-to-no Pfam domain coverage thus cannot be analyzed within this framework. Overall, as gene cluster mining efforts increase in size and scope, there is an urgent need for GCF delineation methods that are scalable, flexible, and generally applicable across gene cluster types.

Here, we present Iterative Gene clUster Analysis (IGUA; https://github.com/zellerlab/IGUA), a scalable, flexible GCF delineation method for genomic segments with multi-gene architectures. Created with large-scale use cases in mind, IGUA outperforms gold-standard GCF delineation tools in terms of time and memory requirements, without sacrificing accuracy. On a BGC clustering task, we show that IGUA is 29× and 10× faster than BiG-SCAPE v2 and BiG-SLiCE v2, respectively (28 CPUs), and to showcase its scalability, we use IGUA to cluster > 2.8 million BGCs from nearly 1 million prokaryotic genomes in under 18 h. Beyond the BGC space, IGUA can be used for many other clustering tasks, as demonstrated for five additional prokaryotic gene cluster types (secretion systems, prophages, genomic islands, phage-plasmids, and CAZyme gene clusters). By delineating GCFs among these large gene cluster datasets, we showcase how the GCF concept can be applied to virtually any type of contiguous gene cluster. Taken together, IGUA represents a versatile GCF delineation tool with unprecedented scalability and flexibility, which can be leveraged in large-scale, data-driven (meta)genomic mining applications.

## Materials and methods

### Implementation of IGUA

IGUA was designed to group gene clusters into GCFs using three major steps: (i) a fragment removal step, followed by (ii) nucleotide-level deduplication and (iii) protein-level clustering steps (Fig. 1). Briefly, (i) the “mmseqs linclust” command in MMseqs2 (at the time of publication, release 15-6f452) [42] identifies and removes fragmented gene clusters, which are substrings of a larger, highly similar gene cluster at the nucleotide (DNA) level, using the following default parameters (i.e. low-coverage/high-identity mode; Fig. 1A): *E*-value = 0.001, sequence identity = 0.85, coverage = 1, cluster mode = 0, coverage mode = 1, spaced *k*-mer mode = 0. After removing gene cluster fragments, (ii) “mmseqs linclust” is used a second time to deduplicate closely related gene clusters at the nucleotide level, using the following default parameters (i.e. high-coverage/medium-identity mode; Fig. 1A): *E*-value = 0.001, sequence identity = 0.6, coverage = 0.5, cluster mode = 0, coverage mode = 0, spaced *k*-mer mode = 0. Both of these nucleotide-level clustering steps account for gene order/synteny when grouping similar gene clusters.

**Figure 1.**
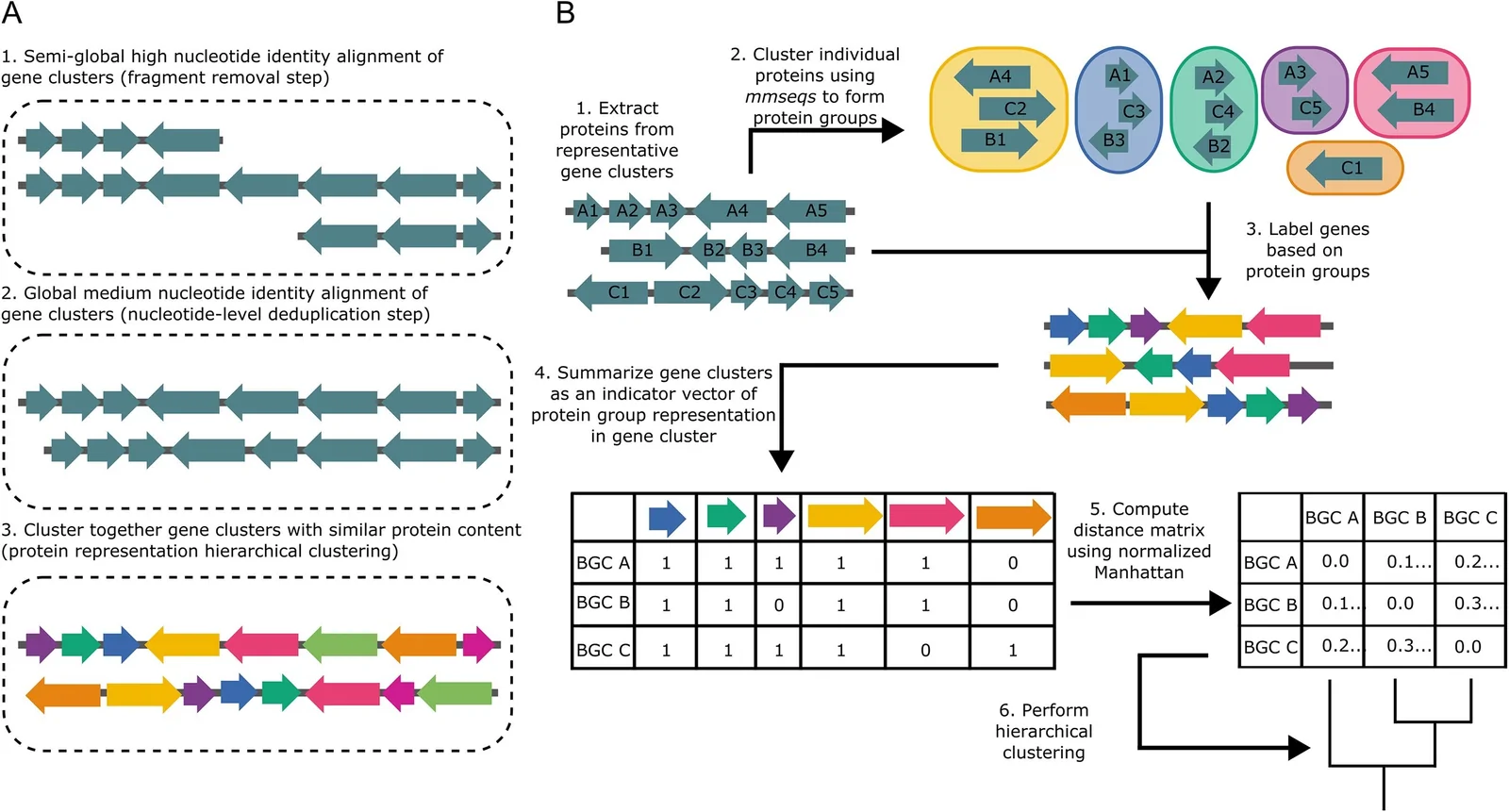
Overview of IGUA’s iterative gene cluster family (GCF) delineation pipeline. (**A**) IGUA clusters gene clusters into GCFs using three major steps: (1) a fragment removal step, (2) a nucleotide (DNA)-level deduplication step, and (3) a clustering step based on protein content, which clusters proteins within representative gene clusters from step (2), irrespective of their order within each gene cluster. (**B**) IGUA’s default protein-level clustering step (step 3) in detail. Users can adjust parameters to e.g. perform linear clustering in lieu of hierarchical clustering, and/or remove clustering weights.

For (iii), the final clustering step based on protein content, all proteins within representative gene clusters from step (ii) are clustered using “mmseqs linclust,” irrespective of their order within each gene cluster (default parameters: *E*-value = 0.001, coverage = 0.9, coverage mode = 1, sequence identity = 0.5), resulting in a set of “protein groups” (Fig. 1A). Protein group counts are then used to populate a sparse protein composition matrix, in which each row represents a gene cluster, each column represents a protein group, and each cell contains the number of proteins in each gene cluster assigned to each protein group (Fig. 1B). The length of each protein group representative is recorded (to be used as weights in the subsequent pairwise distance computation). By default, weighted Manhattan distances are then calculated between all gene clusters using a fast parallel algorithm designed for compressed-sparse-row (CSR) matrices (Fig. 1B). The distances between two clusters are normalized using the sum of their weights (by default; users can alternatively perform unweighted GCF delineation). Gene clusters are then partitioned into GCFs using either (a) hierarchical clustering with the linkage function from the kodama library (at the time of publication, v0.2.0; https://crates.io/crates/kodama) and a user-specified agglomerative clustering method (default) or (b) a linear clustering method, similar to MMseqs2 (Supplementary Text). Together, this results in a set of GCFs, as well as a set of representative gene clusters (one per GCF, i.e. the longest gene cluster from step ii within the GCF; Fig. 1B). Users are able to modify each of the aforementioned parameters as desired.

### Optimization of IGUA parameters on a manually curated dataset

To provide users with optimal default settings for IGUA’s two primary user-specified parameters (i.e. hierarchical clustering linkage method and clustering distance), we used IGUA to cluster a gene cluster dataset with manually curated, ground-truth gene cluster-to-GCF mappings, testing a range of parameter combinations. Briefly, the BGC dataset used to validate the GCF delineation method in the original BiG-SCAPE paper [32] was extracted from MIBiG v1.3 (downloaded from BiG-SCAPE’s GitHub repository; https://github.com/medema-group/BiG-SCAPE/blob/bb2264c8101f8f7204f2799e41aeef2a2505da72/Annotated_MIBiG_reference/MIBiG_1.3_final.zip) [32, 46]; this manually curated dataset consisted of 313 experimentally validated BGCs (gene clusters) associated with 376 chemical compounds grouped into compound groups (corresponding to GCFs) [32]. To mimic the analysis performed in the original BiG-SCAPE paper [32], two of the classes in the manually curated dataset (microcins and lanthipeptides I) were omitted prior to clustering, resulting in a data set consisting of 304 experimentally validated BGCs associated with 366 chemical compounds. To enable comparability between methods, BGCs with >1 GenBank record per file were removed post-clustering, as IGUA treats each GenBank record as a separate BGC, while BiG-SCAPE treats each file as a separate BGC. Altogether, after filtering, a final data set of 292 BGCs associated with 352 chemical compounds was produced (referred to hereafter as the “manually curated dataset”; Supplementary Table S1).

To select optimal default clustering parameters for IGUA, BGCs in the manually curated dataset were clustered into GCFs using IGUA v0.1.0, testing all combinations of the following parameters: (i) linkage method, set to one of “average,” “centroid,” “ward,” “weighted,” “complete,” “median,” “single,” or “linclust” (Supplementary Text); (ii) clustering distance, varied from 0.01 to 1.0 in 0.01 increments (800 total runs representing 800 total parameter combinations). For each IGUA run, singleton GCFs were removed (as was done in the original BiG-SCAPE study, so as to not skew subsequent calculations of GCF purity) [32]. GCFs produced by IGUA were then compared to the manually curated GCFs (the latter representing “ground truth” GCFs) in R v4.1.2 [48], and the following evaluation metrics were calculated: (i) adjusted Rand index (ARI), (ii) adjusted mutual information (AMI), and (iii) normalized mutual information (NMI) scores (aricode package v1.0.3; https://CRAN.R-project.org/package=aricode, accessed 23 December 2024); (iv) purity (funtimes package v9.1; https://CRAN.R-project.org/package=funtimes, accessed 23 December 2024); (v) total number of GCFs. Taking all aforementioned metrics into consideration (as most yielded similar results), a combination of linkage method = “average” and clustering distance = 0.80 was found to yield optimal results (Fig. 2A and Supplementary Fig. S1, Supplementary Text); these parameters were set as the default parameters in IGUA v0.1.0 and used in all subsequent analyses unless otherwise stated.

**Figure 2.**
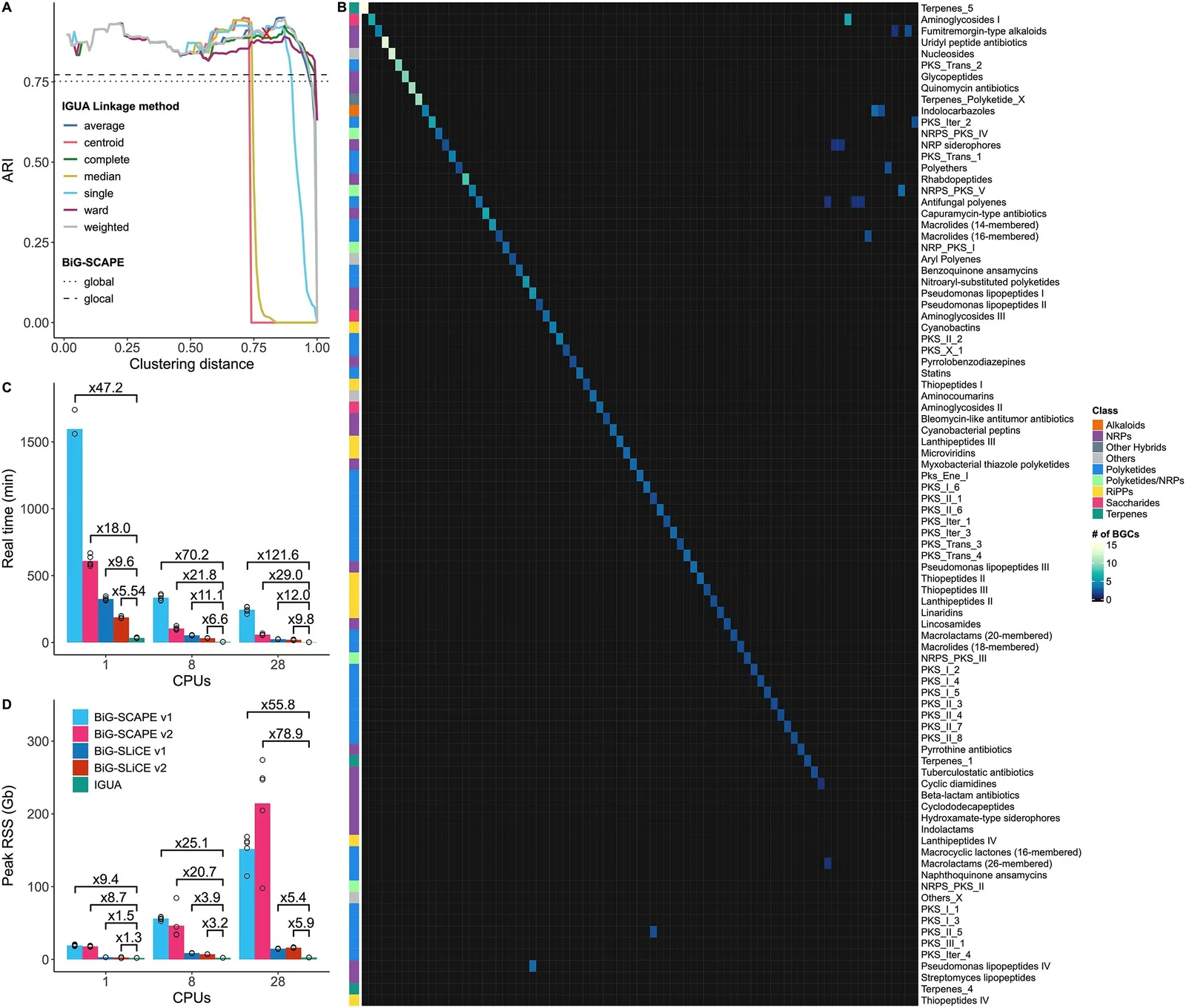
IGUA is faster than the state-of-the-art, without sacrificing accuracy. (**A**) Adjusted Rand index (ARI; *Y*-axis) of IGUA GCFs relative to manually curated, “ground truth” GCFs for each tested parameter combination (clustering distance, *X*-axis; hierarchical clustering linkage method, line color). Horizontal dashed lines denote ARI values achieved by BiG-SCAPE v1 in “global” or “glocal” mode (where a theoretical maximum of 1.0 = the optimal ARI value, corresponding to GCFs that are identical to the ground truth). A clustering distance of 0.80 and the “average” linkage method were selected as IGUA’s (optimal) default parameters (denoted with “X”). For additional clustering metrics, see Supplementary Fig. S1. (**B**) Heatmap comparing manually curated, “ground truth” GCFs (rows) to GCFs delineated using IGUA (linkage method = “average,” clustering distance = 0.80; columns). Cells are shaded according to the number of overlapping BGCs within a GCF. The color strip to the left of the heatmap denotes the biosynthetic class of each manually curated GCF. NRPs, non-ribosomal peptide synthetases; RiPPs, ribosomally synthesized and post-translationally modified peptides. For a larger version of this figure, see Supplementary Fig. S2. (**C**) Average time (real/“wall clock” time, in minutes; *Y*-axis) and (**D**) memory usage (peak resident set size [RSS] in GB; Y-axis) for BiG-SCAPE v1 and v2, BiG-SLiCE v1 and v2, and IGUA, using 1, 8, or 28 CPUs (*X*-axes). For each tool, GCF delineation was carried out using antiSMASH BGCs from five diverse subsets of genomes from the proGenomes3 database (≈5000 genomes each, see text for details). Dots overlapping each bar denote raw values for each proGenomes3 subset.

To evaluate the GCF delineation accuracy of IGUA relative to the state-of-the-art, (i) BiG-SCAPE v1.1.5 (referred to hereafter as BiG-SCAPE v1) [32] and (ii) IGUA v0.1.0 were used to cluster BGCs from the manually curated dataset into GCFs (Supplementary Table S1; note that BiG-SLiCE was not included here, as BiG-SCAPE has already been determined to be more accurate than BiG-SLiCE) [33]. For (i) BiG-SCAPE v1, BGCs were clustered into GCFs in two separate runs, using the following parameters (i.e. the same parameters used in the original BiG-SCAPE v1 paper): hybrid mode disabled (–hybrids-off; both runs), a cutoff value of 0.75 (–cutoffs 0.75; both runs), and either glocal or global alignment mode (–mode glocal or –mode global, run 1 and run 2, respectively; Supplementary Tables S2-S3) [32]. For (ii) IGUA v0.1.0, BGCs were clustered into GCFs using optimal parameters (linkage method = “average” and clustering distance = 0.80, see above; Supplementary Table S4). Post-clustering, singleton GCFs (<2 BGCs) were excluded from further analysis (as was done in the original BiG-SCAPE v1 paper) [32]. BiG-SCAPE v1 and IGUA GCFs were each compared to the manually curated GCFs (representing “ground truth” GCFs) as described above (Fig. 2B, and Supplementary Fig. S2 and Supplementary Table S1).

### Comparison of IGUA and BiG-SCAPE GCFs on a *Streptomyces* dataset

In addition to the manually curated dataset (see section “Optimization of IGUA parameters on a manually curated dataset” above), a dataset consisting of complete *Streptomyces* genomes was used to compare GCFs delineated by IGUA to those delineated by BiG-SCAPE v1 (similar to the *Streptomyces* dataset used in the original BiG-SCAPE v1 paper; note that the exact *Streptomyces* dataset described in the BiG-SCAPE v1 paper could not be used, as not all genomes/accession numbers were provided) [32]. Briefly, GenBank assemblies for all complete *Streptomyces* genomes in NCBI’s Assembly database [49] were downloaded (*n* = 492 complete genomes, accessed 9 December 2023). antiSMASH v6.1.1 [50] was used to detect BGCs in each *Streptomyces* genome, using Prodigal in metagenomic mode as the genefinding tool (–genefinding-tool prodigal-m) and two CPUs (-c 2; remaining parameters were set to their defaults) [51]. The resulting antiSMASH BGCs (*n* = 15 298) were clustered into GCFs using: (i) BiG-SCAPE v1.1.5, with “glocal” mode (–mode glocal), singletons included (–include_singletons), hybrid mode disabled (–hybrids_off), and MIBiG v3.1 included (–mibig) [52]; (ii) IGUA v0.1.0, which was run with optimal parameters (linkage method = “average” and clustering distance = 0.80) and MIBiG v3.1 BGCs included (*n* = 17 800 BGCs total). AMI, NMI, and ARI were calculated relative to BiG-SCAPE v1 clustering results as described above (see section “Optimization of IGUA parameters on a manually curated dataset”; Supplementary Fig. S3, Supplementary Tables S5 and S6).

To further assess IGUA’s speed relative to input dataset size, the full set of *Streptomyces* antiSMASH BGCs (with MIBiG v3.1 BGCs included) was down-sampled to (i) 1000, (ii) 2500, (iii) 5000, and (iv) 10 000 BGCs in triplicate (*n* = 12 total down-sampled *Streptomyces* datasets) using the random.sample() function from the random module of Python v3.10.4. Each down-sampled *Streptomyces* dataset was clustered using IGUA v0.1.0 (8 CPUs and optimal parameters, i.e. linkage method = “average” and clustering distance = 0.80), with runtime logged using Nextflow v24.04.2 (i.e. by enabling the “report” option in Nextflow’s configuration file; Supplementary Fig. S4, Supplementary Tables S7 and S8) [53].

### Speed and memory benchmarking using species representative genomes

To evaluate speed and memory requirements for each of BiG-SCAPE v1.1.5 [32], BiG-SLiCE v1.1.0 [33], and IGUA v0.1.0, BGCs from five diverse subsets of genomes from the proGenomes3 database [31] were used. Briefly, antiSMASH v6.1.1 was used to detect BGCs in all proGenomes3 representative genomes as described above (*n* = 41 777 genomes; see section “Comparison of IGUA and BiG-SCAPE GCFs on a *Streptomyces* dataset”). Genome Taxonomy Database (GTDB) taxonomic assignments [54] for all proGenomes3 representative genomes were downloaded from proGenomes3 (http://progenomes3.embl.de/data/proGenomes3_specI_lineageGTDB.tab.bz2; accessed 17 October 2022) [54, 55]. To ensure that each subset of the proGenomes3 representative genomes contained diverse, non-redundant genomes, one specI cluster (i.e. species) [55] per GTDB genus was sampled, and from this, 5000 specI clusters were randomly selected. For each randomly selected specI cluster, the specI representative genome (per proGenomes3) was selected. This process was repeated five times to generate five samples of approximately 5000 genomes each (some specI clusters lacked representative genomes; in these rare cases, the specI cluster was not included in the subset). Overall, this resulted in five subsets, each containing antiSMASH BGCs from ≈ 5000 diverse genomes, each representing a distinct prokaryotic genus (Supplementary Table S9).

For each of the five subsets, all antiSMASH BGCs from the sampled representative genomes were clustered with (i) BiG-SCAPE v1.1.5, (ii) BiG-SLiCE v1.1.0 (referred to hereafter as BiG-SLiCE v1), and (iii) IGUA v0.1.0. For (i) BiG-SCAPE v1, antiSMASH BGCs were clustered alongside BGCs from the MIBiG database (–mibig; v3.1), using glocal mode (–mode glocal), the “include singletons” option enabled (–include_singletons), and “hybrid mode” disabled (–hybrids-off). For (ii) BiG-SLiCE v1, default settings were used to cluster all antiSMASH BGCs into GCFs (MIBiG BGCs were not included, as BiG-SLiCE v1 did not accept MIBiG-formatted BGCs). For (iii) IGUA, antiSMASH BGCs detected in each subset of genomes were concatenated with BGCs from MIBiG v3.1; IGUA was then used to cluster each concatenated subset using optimal settings (linkage method = “average” and clustering distance = 0.80). For each of the five subsets, all three methods were used to cluster BGCs into GCFs using 1, 8, or 28 CPUs. For each run, runtime and memory requirements were logged by enabling the “report” option in the Nextflow configuration file (Fig. 2C,D; Supplementary Table S10).

The aforementioned speed and memory benchmarking steps were additionally repeated using BiG-SCAPE v2.0.0 (parameters: –mix –hybrids-off –include-singletons –pfam-path [path to Pfam file]) and BiG-SLiCE v2.0.2 (parameters: –threshold 300 –program_db_folder [path to BiG-SLiCE models file]), which were published while this manuscript was under review (referred to hereafter as BiG-SCAPE v2 and BiG-SLiCE v2, respectively) [34]. For the BiG-SCAPE v2 runs, antiSMASH-processed MIBiG BGCs were downloaded (v3.1, accessed 26 November 2025; https://dl.secondarymetabolites.org/mibig/) and used. Akin to BiG-SLiCE v1, BiG-SLiCE v2 did not recognize these antiSMASH-processed MIBiG BGCs, and they were hence not included in the analysis. In all instances besides BiG-SLiCE v2 with 8 CPUs (which was allocated 8 CPUs), 28 CPUs were allocated to each clustering job, while the actual process ran with the specified number of CPUs (i.e. 1, 8, or 28 CPUs).

### Clustering of >2.8 million GECCO BGCs from >900 000 prokaryotic genomes

To showcase its speed and scalability, IGUA was used to cluster all BGCs detected in the proGenomes3 database [31] into GCFs. Briefly, GenBank records for all BGCs identified in all proGenomes3 genomes were downloaded (*n* = 2 826 569 total BGCs from 907 388 total genomes, detected using GECCO, a ML-based BGC detection method, as described previously; http://progenomes3.embl.de/data/progenomes3_gecco_clusters.gbk.gz) [23, 31]. IGUA v0.1.0 was used to cluster all 2 826 569 BGCs, plus 2502 experimentally validated BGCs from MIBiG v3.1, into GCFs (using 72 CPUs, linkage method = “average,” and clustering distance = 0.80; Supplementary Table S11). Runtime and memory usage were logged by enabling the “report” option in the Nextflow configuration file.

To visualize proGenomes3 GCFs in two-dimensional space, a UMAP [56] was constructed using GCF representatives output by IGUA (*n* = 56 960 GCF representative BGCs). To construct the UMAP, pyHMMER v0.8.2 [57] was used to annotate each IGUA GCF representative BGC (28 CPUs and default settings), using Pfam v35.0 profile hidden Markov models (pHMMs; downloaded from the Pfam FTP site (https://ftp.ebi.ac.uk/pub/databases/Pfam/releases/Pfam35.0/Pfam-A.hmm.gz) [47]. From this, a Pfam domain composition matrix was computed (via https://zenodo.org/records/21976829/files/umap_bgcs.py?download=1) and extracted for UMAP construction. The UMAP was produced in R v4.3.1, using the packages Seurat v5.0.2 [58], Signac v1.12.0 [59], reticulate v1.35.0 (https://CRAN.R-project.org/package=reticulate, accessed 23 December 2024), and clustree v0.5.1 [60] (see Supplementary Text for details; Supplementary Fig. S5 and Supplementary Table S12).

### Clustering of secretion system clusters

To showcase its utility beyond BGC clustering, we used IGUA to cluster five additional prokaryotic gene cluster types (secretion systems, prophages, genomic islands, phage-plasmids, and CAZyme gene clusters) into GCFs. For secretion system clustering, metadata for all secretion systems in TXSSdb [10] (i.e. nucleotide accession numbers, start/end coordinates, and secretion system type classifications) were downloaded (*n* = 11 939 total secretion system entries; http://macsydb.web.pasteur.fr/txssdb/_design/txssdb/index.html, accessed 27 September 2024). Secretion systems classified by TXSSdb as “single_locus” secretion systems were used in subsequent steps (*n* = 10 784 TXSSdb single-locus secretion systems). The “entrez_fetch” command in the rentrez package (v1.2.3) [61] in R v4.3.2 was used to query NCBI’s Nucleotide database [62] for each single-locus secretion system, using NCBI Nucleotide accession numbers and secretion system start/end coordinates provided by TXSSdb. This resulted in a total of 10 576 valid GenBank files (one per single-locus secretion system), which were concatenated into a single GenBank file using BioPython v1.84 [63]. IGUA v0.1.0 was then used to cluster all 10 576 TXSSdb single-locus secretion systems into GCFs (linkage method = “average,” clustering distance = 0.80; Supplementary Table S13). The “entrez_search” and “entrez_summary” commands in rentrez were further used to download the host bacterium genome for each secretion system, and each host bacterium genome underwent taxonomic classification using GTDB release214/GTDB-Tk v2.3.2 (i.e. via GTDB-Tk’s “classify_wf” workflow with –cpus 28 and default settings; Supplementary Table S14) [54, 64]. A UMAP of the IGUA GCF representative secretion systems was constructed (see Supplementary Text for details; Supplementary Fig. S6 and Supplementary Table S15).

### Clustering of prophage clusters

For prophage clustering, all prophage sequences in Prophage-DB [30] were downloaded (i.e. bacterial_host_prophages.tar.gz, archaeal_host_prophages.tar.gz, unknown_host_prophages.tar.gz, and all corresponding metadata; https://doi.org/10.5061/dryad.3n5tb2rs5, accessed 26 November 2024). Prokka v1.14.6 [65] was used to annotate each prophage using its respective FASTA file as input, the “Viruses” database (–kingdom Viruses), and the “–addgenes” and “–compliant” options enabled (remaining options were set to their defaults). Annotated GenBank files produced by Prokka (*n* = 356 776 files, one per Prophage-DB prophage) were concatenated into a single GenBank file and clustered into GCFs using IGUA (linkage method = “average,” clustering distance = 0.80; Supplementary Table S16). This resulted in a large number of GCFs (*n* = 213 699); to reduce the number of GCFs further (for dataset visualization purposes), IGUA was run a second time, using average linkage and a higher clustering distance threshold (i.e. 0.99, resulting in 106 749 GCFs; Supplementary Table S17). IGUA GCF representatives identified using this higher threshold (0.99) were visualized via a UMAP (see Supplementary Text for details; Supplementary Fig. S7 and Supplementary Table S18).

### Clustering of genomic islands

For genomic island clustering, each individual IslandViewer 4 dataset (i.e. genomic islands detected using one of four different methods: IslandPick, IslandPath-DIMOB, SIGI-HMM, or Islander) was downloaded as a tab-separated (TSV) file (downloaded 8 November 2025; see Supplementary Text for details) [7, 66–69]. The “entrez_fetch” command in the rentrez package (v1.2.3) [61] in R v4.3.2 was used to query NCBI’s Nucleotide database [62] for each genomic island, using NCBI Nucleotide accession numbers and genomic island start/end coordinates provided by IslandViewer 4. This resulted in a total of 902 158 valid GenBank files (one per genomic island), which were concatenated into a single GenBank file using BioPython v1.84 [63]. IGUA v0.1.0 was then used to cluster all 902 158 genomic islands into GCFs (linkage method = “average,” clustering distance = 0.80; Supplementary Table S19). A UMAP of the IGUA GCF representative genomic islands was constructed (see Supplementary Text for details; Supplementary Figs S8 and S9, Supplementary Table S20).

### Clustering of phage-plasmids

For phage-plasmid clustering, Supplementary Dataset S1 from Pfeifer and Rocha [70], which contains NCBI Nucleotide accession numbers for a selection of phage, plasmids, and phage-plasmids, was downloaded (accessed 12 December 2025). The “entrez_fetch” command in the rentrez package (v1.2.3) [61] in R v4.3.2 was used to query NCBI’s Nucleotide database [62] for each phage/plasmid/phage-plasmid, using the NCBI Nucleotide accession number for each. This resulted in a total of 25 197 valid GenBank files (one per each individual phage/plasmid/phage-plasmid), which were concatenated into a single GenBank file using BioPython v1.84 [63]. IGUA v0.1.0 was then used to cluster all 25 197 phage/plasmids/phage-plasmids into GCFs (linkage method = “average,” clustering distance = 0.80; Supplementary Table S21). A UMAP of the IGUA GCF representative phage/plasmids/phage-plasmids was constructed (see Supplementary Text for details; Supplementary Figs S9, S10, and Supplementary Table S22).

### Clustering of CAZyme gene clusters

To construct a large dataset of CAZyme (carbohydrate active enzyme) gene clusters, the latest version of MGnify’s “human-gut” database (v2.0.2) was downloaded via the MGnify FTP site, resulting in a set of 289 232 microbial genomes and metagenome-assembled genomes (MAGs) with associated metadata; see Supplementary Text for details) [71]. The FASTA file for each genome/MAG (*n* = 289 232) was then supplied as input to run_dbCAN v5.2.1 [72], which was used to detect CAZyme gene clusters using the easy_CGC workflow (see Supplementary Text for details) [72]. The resulting genome/MAG GFF file, plus the CAZyme gene cluster coordinate file (both produced by run_dbCAN) were then supplied as input to gff2gbk v0.1.0 (https://github.com/lmc297/gff2gbk; -i and -r, respectively), which was used to extract the CAZyme gene cluster from the GFF file and convert it to a GenBank file (see Supplementary Text for details).

Overall, the above approach resulted in a total of 9 010 369 valid GenBank files (one per CAZyme gene cluster); these GenBank files were concatenated into a single GenBank file alongside the 579 experimentally characterized CAZyme gene clusters from the dbCAN-PUL database [73] with valid GenBank files (https://pro.unl.edu/static/DBCAN-PUL/, accessed 8 November 2025) using BioPython v1.84 [63]. IGUA v0.1.0 was then used to cluster all 9 010 948 CAZyme gene clusters into GCFs (linkage method = “average,” clustering distance = 0.80; Supplementary Table S23). A UMAP of the IGUA GCF representative CAZyme gene clusters was then constructed (see Supplementary Text for details; Supplementary Figs S9 and S11, Supplementary Table S24).

### Data visualization

Plots were constructed in R v4.3.1, using the following packages (all URLs accessed 23 December 2024): readxl v1.4.3 (https://CRAN.R-project.org/package=readxl), tidyverse v2.0.0 [74], khroma v1.13.0 (https://packages.tesselle.org/khroma/), ggnewscale v0.4.10 (https://CRAN.R-project.org/package=ggnewscale), ggpubr v0.6.0 (https://CRAN.R-project.org/package=ggpubr), ComplexHeatmap v2.18.0 [75], ggplotify v0.1.2 (https://CRAN.R-project.org/package=ggplotify), ggalluvial v0.12.5 (http://corybrunson.github.io/ggalluvial/), ggmagnify v0.4.1.9000 (https://github.com/hughjonesd/ggmagnify), ggsignif v0.6.4 [76], Signac v1.12.0 [59], Seurat v5.0.2 [58], and clustree v0.5.1 [60]. A plot comparing secretion system gene clusters was generated using clinker v0.0.31 [77], with default settings.

## Results

### IGUA: accurate, scalable GCF delineation via iterative clustering

The IGUA pipeline is composed of three major steps, which are designed to tackle three major problems associated with GCF delineation (Fig. 1). The first step, (i) a nucleotide-level fragment removal step, uses MMseqs2 in low-coverage/high-identity mode to remove sequences that are fragments (subsequences or substrings) of a larger gene cluster (Fig. 1A). This reduces computation time/memory requirements needed in later steps, particularly for metagenomic datasets, which are often composed of highly redundant, fragmented MAGs.

The second step, (ii) nucleotide-level deduplication, uses MMseqs2 in high-coverage/medium-identity mode to group highly similar (e.g. nearly identical) gene clusters together, producing a set of representative gene clusters (Fig. 1A). This further reduces computation time/memory requirements needed in the third step, (iii) protein-level clustering. In this final step, all proteins within all gene clusters undergo unsupervised clustering (irrespective of their position within the gene cluster) via MMseqs2 to produce protein groups. The resulting protein groups are then used to produce a sparse matrix, capturing the protein content of each gene cluster. In this sparse matrix, each gene cluster is encoded as a vector of protein group representation (Fig. 1B). By default, pairwise distances between gene clusters are computed using weighted and normalized Manhattan distances, in order to account for variability in protein length and total cluster length between each cluster pair (Fig. 1B). In other words, the distance computation between gene clusters is weighted using the length of each protein group representative; this ensures that the absence of a larger gene (e.g. for BGCs, a core biosynthetic gene in a non-ribosomal peptide synthetase) is weighted more than the absence of a smaller gene (e.g. a regulatory gene; note that users can optionally use unweighted distances). Finally, hierarchical clustering is used to delineate gene clusters into GCFs (Fig. 1). Altogether, IGUA’s iterative clustering approach removes redundancy early on (i.e. in the fragment removal and nucleotide-level deduplication steps) to reduce time and memory usage later (i.e. in the more computationally intensive, protein-level clustering step; Fig. 1).

### IGUA outperforms the state-of-the-art in terms of compute time and memory usage, without sacrificing accuracy

IGUA primarily uses two user-specified parameters for GCF delineation: (i) linkage method (for hierarchical clustering/dendrogram construction), and (ii) clustering distance (the distance at which the dendrogram is “cut” to adjust the granularity of the resulting GCFs; Fig. 1B). To provide users with optimal default parameters, we applied IGUA to a manually curated dataset with “ground truth” gene cluster-to-GCF mappings (i.e. the BGC-to-GCF dataset used to benchmark and validate BiG-SCAPE) [32], testing a range of parameter combinations. After considering GCF composition/purity and the total number of GCFs produced, we selected (i) average linkage hierarchical clustering with (ii) a clustering distance threshold of 0.80 as the optimal default parameter combination for IGUA (Fig. 2A and Supplementary Fig. S1; see section “Optimization of IGUA parameters on a manually curated dataset” above). However, aside from several linkage methods that produced suboptimal results (e.g. centroid, median, and single), a relatively wide range of parameter combinations produced (near-)optimal results, indicating that GCF delineation among BGCs is robust to parameter selection (Fig. 2A and Supplementary Fig. S1). Notably, using these selected parameters—but also across a wide range of other parameter settings—IGUA performed slightly better than BiG-SCAPE v1 on this manually curated, “ground truth” BiG-SCAPE validation dataset (Fig. 2B, and Supplementary Fig. S2, Supplementary Tables S2–S4). On the *Streptomyces* dataset (i.e. a set of 15 298 antiSMASH BGCs from 492 complete *Streptomyces* genomes, similar to the *Streptomyces* dataset used in the BiG-SCAPE v1 publication), both IGUA and BiG-SCAPE v1 produced similar GCFs (NMI = 0.83 and 0.82 with singleton GCFs included and removed, respectively; Supplementary Fig. S3, Supplementary Tables S5 and S6, see section “Comparison of IGUA and BiG-SCAPE GCFs on a *Streptomyces* dataset” above). Overall, these benchmarks showed IGUA’s GCF delineation accuracy to be on par with the state-of-the-art for BGC clustering tasks.

Beyond validating the accuracy of IGUA’s GCF delineation method, we further benchmarked IGUA’s speed and memory usage relative to BiG-SCAPE v1 and v2 (the state-of-the-art methods in terms of accuracy) and BiG-SLiCE v1 and v2 (the state-of-the-art methods in terms of speed; Fig. 2C,D). To accomplish this, each of the five methods (BiG-SCAPE v1, BiG-SCAPE v2, BiG-SLiCE v1, BiG-SLiCE v2, and IGUA) was used to cluster five sets of BGCs from ≈ 5000 diverse prokaryotic genomes (each representing a unique genus) into GCFs, using 1, 8, or 28 CPUs (Fig. 2C,D and Supplementary Table S10). Using 1 CPU, IGUA was 47× and 10× faster on average than BiG-SCAPE v1 and BiG-SLiCE v1, and 18× and 6× faster on average than BiG-SCAPE v2 and BiG-SLiCE v2, respectively; as the number of CPUs increased, the performance gap increased, with IGUA becoming an order of magnitude faster than BiG-SLiCE v2 at 28 CPUs (Fig. 2C). Similar results were observed for memory usage (Fig. 2D and Supplementary Table S10). Taken together, IGUA represents an accurate GCF delineation method, which is faster than the state-of-the-art and more memory efficient.

### IGUA readily scales to millions of biosynthetic gene clusters

To showcase the speed, scalability, and flexibility of IGUA, we delineated GCFs among >2.8 million BGCs, detected in nearly 1 million prokaryotic genomes using GECCO, a ML-based BGC detection method [23, 31]. Using 72 CPUs, IGUA clustered 2 829 071 BGCs (2 826 569 GECCO BGCs detected in 907 388 genomes from the proGenomes3 database, plus 2502 experimentally validated BGCs from the MIBiG database) [31] into 56 960 GCFs in 17 h and 22 min (Fig. 3A–C). To visualize these results in two-dimensional space, we constructed a UMAP using the protein domain composition of GCF representative BGCs identified by IGUA, and we used Seurat clustering to identify larger GCF clusters within the UMAP (Fig. 3A).

**Figure 3.**
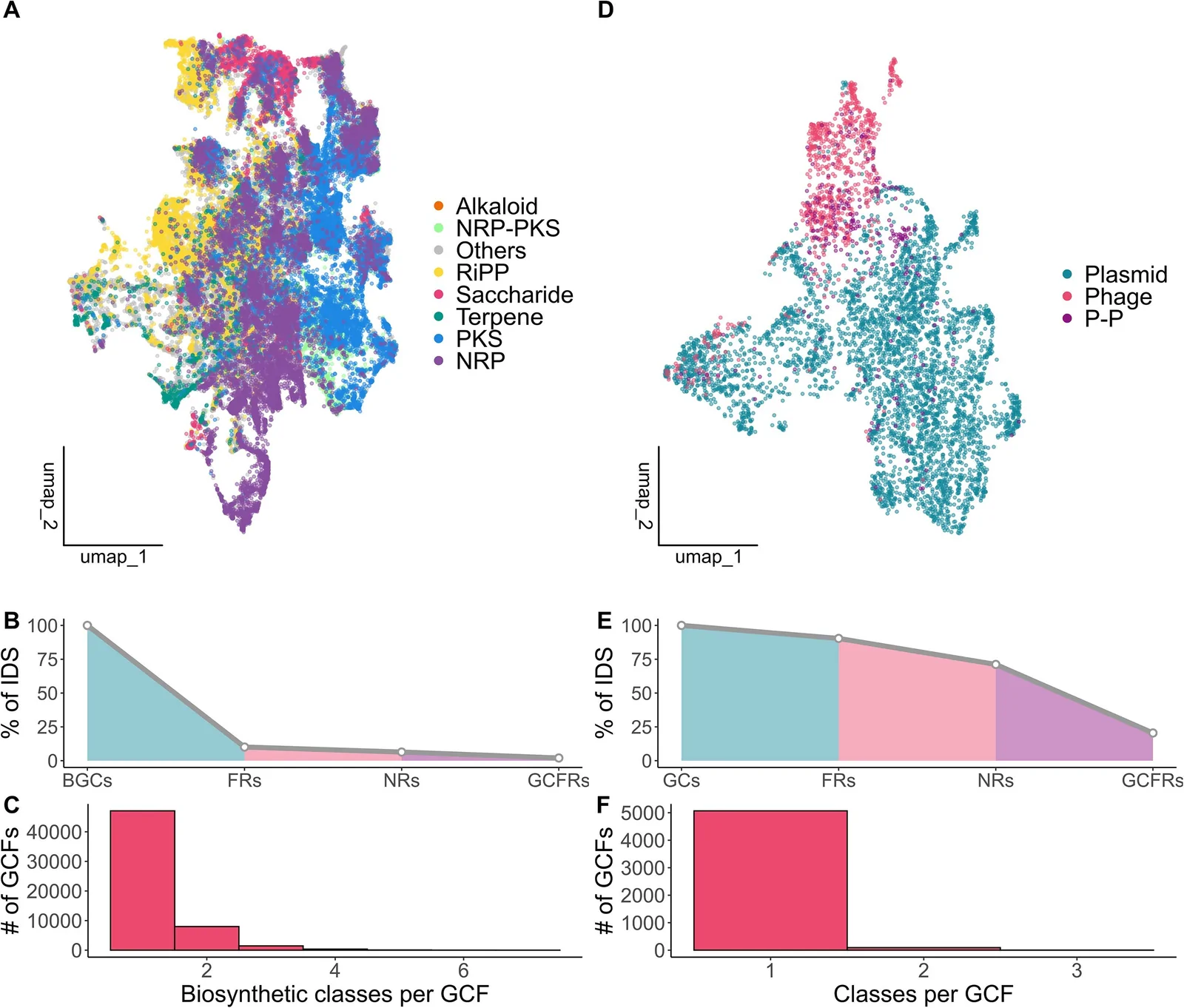
IGUA is a flexible GCF delineation tool, which can scale to massive datasets. IGUA was used to cluster a variety of gene clusters into GCFs, including (**A–C**) BGCs (*n* = 2 829 071 BGCs from the proGenomes3 and MIBiG databases) [31, 52] and (**D–F**) plasmids, phage, and phage-plasmids (P–P; *n* = 25 197 total gene clusters compiled in a previous study) [70]. Plots denote the following (from top to bottom): gene cluster UMAPs of IGUA GCF representatives (points), colored by GCF majority (A) predicted biosynthetic class (for BGCs) or (D) gene cluster type (i.e. one of plasmid, phage, or P–P); (B and E) area plots showing the reduction in data set size (% of initial data set size [IDS], *Y*-axes) at each step in IGUA’s pipeline (*X*-axes). (B) BGCs and (E) gene clusters (GCs) correspond to the original input data set (i.e. 100% of the IDS), while FRs (fragment representatives), NRs (nucleotide representatives), and GCFRs (GCF representatives) correspond to the percentage of the input data set maintained at steps i (fragment removal), ii (nucleotide-level deduplication), and iii (protein-level clustering) in the IGUA pipeline, respectively; (C and F) histograms showing the number of (C) biosynthetic classes and (D) gene cluster types (plasmid, phage, or P–P; *X*-axes) spanned by each GCF (*Y*-axes). To view IGUA results for four additional prokaryotic gene cluster datasets (secretion systems, prophages, genomic islands, and carbohydrate active enzyme [CAZyme] gene clusters), see Supplementary Fig. S9.

Within this large BGC dataset, the majority of GCFs (47 069 of 56 960 GCFs, including singletons; 82.6%) consisted of BGCs of a single biosynthetic class (Fig. 3C). Further, an overwhelming proportion of the resulting GCFs did not contain any experimentally validated MIBiG BGCs (55 078 of 56 960 total GCFs, 96.7%), indicating that most areas of the prokaryotic biosynthetic landscape have never been queried experimentally (Fig. 3A–C, and Supplementary Fig. S5, Supplementary Tables S11 and S12). Overall, IGUA can readily scale to massive datasets consisting of millions of BGCs, including BGCs detected using newer ML methods.

### IGUA can be used with any type of prokaryotic gene cluster

IGUA’s iterative clustering approach is agnostic to protein function. Unlike other GCF delineation tools, it does not rely on the presence of specific protein domains and accommodates multiple input types. As such, IGUA can be used for GCF delineation tasks beyond BGC clustering and on proteins that are not well annotated with Pfam domains. To demonstrate this, we used IGUA to delineate GCFs among five additional types of prokaryotic gene clusters (secretion systems, prophages, genomic islands, phage-plasmids, and carbohydrate active enzyme [CAZyme] gene clusters), and we constructed UMAPs and performed GCF representative Seurat clustering for each. We discuss two types (phage-plasmids and secretion systems) in detail below (to view and compare IGUA results for all six prokaryotic gene cluster datasets, see Supplementary Fig. S9).

For the phage-plasmids dataset, IGUA clustered 20 197 plasmids, 3585 phages, and 1415 phage-plasmids from a previous study [70] (*n* = 25 197 total gene clusters) into 5165 GCFs in 43 min using 72 CPUs (Fig. 3D–F, and Supplementary Figs S9, S10, and Supplementary Tables S21 and S22). An overwhelming majority of the resulting GCFs (5068 of 5165 GCFs, including singletons; 98.1%) consisted of only phage, only plasmids, or only phage-plasmids (Fig. 3F). Compared to the proGenomes3 BGC dataset, which reduced to 2.0% of its original size (i.e. 28 29 071 BGCs to 56 960 GCFs; Fig. 3B), the phage-plasmid dataset was considerably more diverse, with IGUA reducing the dataset to 20.5% of its original size (Fig. 3E and Supplementary Table S21). Notably, a UMAP constructed using the protein domain composition of the resulting GCF representative gene clusters revealed a continuum between plasmids, phage-plasmids, and phage (Fig. 3D), mirroring results observed in the dataset’s original study, produced using a different method (i.e. a sequence similarity network) [70].

For the secretion system dataset, IGUA clustered all 10 576 single-locus secretion systems from TXSSdb [10] into 2744 GCFs in under 3 min using 28 CPUs (Fig. 4, Supplementary Fig. S6, and Supplementary Tables S13–S15). In terms of their taxonomic spread, secretion system GCFs tended to retain a taxonomic footprint, as the vast majority were confined to a single phylum (2421 of 2744 GCFs, 88.2%, including singletons; Supplementary Fig. S9, Supplementary Tables S13 and S14). Of all 2744 GCFs, 2639 (96.2%) contained a single secretion system type (singleton GCFs included; Supplementary Table S13).

**Figure 4.**
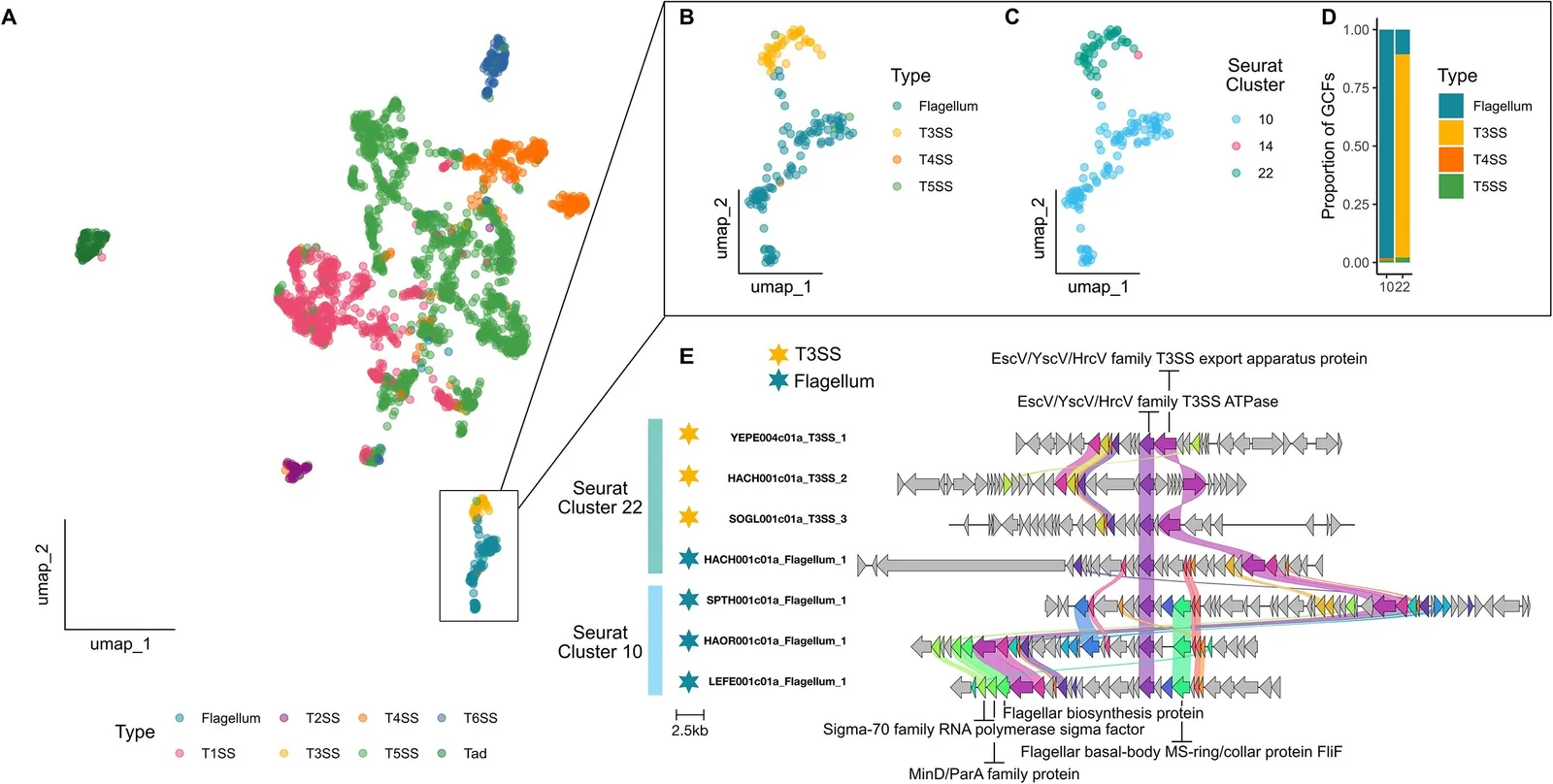
Seurat clusters of IGUA GCF representatives capture a conserved Type III secretion system (T3SS) and flagellar genomic architecture. (**A**) UMAP of secretion system GCF representatives (dots), colored by majority secretion system type per GCF. (**B, C**) UMAP of the T3SS and flagellar Seurat clusters, colored by (B) majority secretion system type per GCF, and (C) Seurat clusters at a resolution of 0.8. (**D**) Proportions of GCF representative secretion system types per Seurat clusters 10 and 22, at a resolution of 0.8. (**E**) Comparison of T3SS (yellow stars) and flagellar (blue stars) secretion systems from Seurat clusters 22 (top four secretion systems) and 10 (bottom three secretion systems). The plot was constructed using clinker [77], with genes represented by arrows and linkages between them to indicate amino acid (AA) identity (AA identity threshold = 0.3).

Within the secretion system UMAP, a total of 17 Seurat clusters could be discerned (Seurat clustering resolution = 0.35; Fig. 4 and Supplementary Table S15). While some secretion system types were distributed across the UMAP (e.g. T5SS, T1SS to a lesser extent), others tended to be confined to a particular Seurat cluster (e.g. T2SS, T6SS; Fig. 4A). Notably, using Seurat clustering, T3SS clustered together with flagellar gene clusters (Fig. 4A, B, and Supplementary Fig. S6), which is consistent with previous reports of a shared T3SS/flagellar genomic architecture and evolutionary history [78–80]. Upon increasing the Seurat clustering resolution to the default 0.80, T3SS and flagellar gene clusters were split into two distinct, nearly pure Seurat clusters (98.1% of 108 GCF representatives in Seurat cluster 10 being flagellar, 87.2% of 47 GCF representatives in Seurat cluster 22 being T3SS; Fig. 4C,D). While examining the gene content of T3SS and flagellar GCF representatives, we noted that T3SS type export apparatus proteins and ATPase were shared among the secretion systems (Fig. 4E). However, the flagellar GCF representatives additionally included flagellar basal-body proteins and biosynthesis proteins, which were absent among the T3SS GCF representatives (Fig. 4E), providing further support for the observed Seurat clustering.

The secretion system UMAP further indicated that “tight adherence” (Tad) secretion systems formed a separate Seurat cluster, which was distinct from other T4SS (Fig. 4A). It has been hypothesized that bacterial Tad secretion systems were acquired from archaea via horizontal gene transfer [81–83], and our results are consistent with previous literature suggesting that Tad secretion systems are distinct from other bacterial secretion systems, including T4SS and type IV pili (T4P) [83]. Overall, these results demonstrate that the GCF concept can be applied to biological problems beyond the BGC space. By generalizing GCF delineation, IGUA can provide novel insights into gene cluster ecology and evolution.

## Discussion

Improvements in data generation methods, computing hardware, gene cluster annotation software, and data storage/management practices are allowing gene cluster mining efforts to target larger datasets than ever before [28, 31, 84]. Scalable GCF delineation methods are thus crucial for making sense of these increasingly massive gene cluster collections [32–34]. Here, we demonstrate that IGUA, our iterative GCF delineation method, has several advantages over previous methods designed specifically for BGC clustering. Most notably, we showed that IGUA is an order of magnitude faster than BiG-SLiCE v2 (28 CPUs), with comparable accuracy to BiG-SCAPE (the state-of-the-art methods in terms of speed and accuracy, respectively) [32–34]. We further showcase IGUA’s (i) scalability, by delineating GCFs among >2.8 million BGCs (>2× the size of the dataset queried in the BiG-SLiCE v1 publication) [33], and (ii) flexibility, by applying IGUA to five additional types of multi-gene clusters.

While IGUA is well-suited for large-scale gene cluster mining efforts, it is important to point out its limitations and outline scenarios/applications where users may prefer alternatives. First, because it is agnostic to gene cluster type, IGUA lacks some of the BGC-specific features that users of BiG-SCAPE and BiG-SLiCE are accustomed to (e.g. a command-line option to cluster MIBiG BGCs alongside user-supplied antiSMASH BGCs; graphical/interactive output files). However, because IGUA can use antiSMASH GenBank files and zip archives as input, we expect IGUA to be accessible to most antiSMASH users, particularly those working with large datasets. Second, by default, IGUA’s protein-level clustering step (step 3) incorporates a final hierarchical clustering step for GCF delineation, which is, in a worst-case scenario, quadratic in time and memory (Fig. 1B). To overcome this limitation, users can employ IGUA’s linear protein clustering method (i.e. “–clustering-method linclust”), which, by most metrics, performs similarly to the default clustering method (Supplementary Fig. S1), while scaling linearly with input dataset size (Supplementary Text). Finally, IGUA’s performance boundaries depend on how much memory and storage a user has available to them (typically in line with MMseqs2 expectations) [42], as well as the dataset being clustered (e.g. the size and diversity of input gene clusters). For example, using 28 CPUs, IGUA took under 3 min to cluster >10k single-locus secretion systems. The highly diverse phage-plasmid dataset, which was roughly only 2× as large, took >14× as long to cluster using 72 CPUs, largely because the first two redundancy removal steps were not as effective on such highly diverse sequences. Thus, performance is dataset-specific and may vary depending on the compute/storage resources available.

Overall, IGUA represents a versatile GCF delineation tool with unmatched computational efficiency and flexibility, enabling (meta)genomic mining applications at unprecedented scales. We anticipate that IGUA will be particularly useful for e.g. redundancy removal (particularly among gene clusters detected in highly fragmented MAGs), functional prediction (e.g. comparing gene clusters of unknown function to gene clusters with known functions), novel gene cluster discovery/prioritization, and GCF delineation among gene cluster types that lack specialized tools.

## Supplementary Material

gkag975_Supplemental_Files

## Data Availability

IGUA is open-source and freely available via GitHub under the GPL-3.0 License (https://github.com/zellerlab/IGUA). Source code for the IGUA version used in the manuscript (v0.1.0, with default parameters set to the optimal values identified in this study) is available via Zenodo (https://doi.org/10.5281/zenodo.15348788). Source and compiled releases are available to download from the Python Package Index (PyPI, https://pypi.org/project/igua/) and from Bioconda (https://anaconda.org/bioconda/igua) [85]. Accession numbers of genomes used in the study, as well as all benchmarking and validation results generated in the study, are available as Supplementary Data. Analysis code is available via Zenodo under DOI: 10.5281/zenodo.21976829 (https://doi.org/10.5281/zenodo.21976829), including a notebook demonstrating how to create figures in the IGUA manuscript.

## Supplementary data

Supplementary data is available at NAR online and via Zenodo. Specifically, Supplementary Figures and Text are available at NAR Online, while other Supplementary Data, including all Supplementary Tables, are available via Zenodo under DOI: 10.5281/zenodo.21976829 (https://doi.org/10.5281/zenodo.21976829).
