# Supplementary material for: Fast, flexible gene cluster family delineation with IGUA
Source: Nucleic Acids Res. 2026 Oct 13;54(19):gkag975. doi: 10.1093/nar/gkag975 (PMC13659240; doi:10.1093/nar/gkag975)
Supplement: Supplementary file 1 [file gkag975_supplemental_files.zip › SupplementaryText_v1.pdf]

Supplementary Text

for

Fast, flexible gene cluster family delineation with IGUA

Supplementary Methods

Implementation and benchmarking of the linclust method in IGUA v0.2.0. By default, IGUA uses a hierarchical clustering step to delineate gene cluster families (GCFs), a step that is quadratic in time and memory *in the worst case* (although, as demonstrated here, often affordable in practice). In an attempt to speed IGUA up further, we implemented a linear clustering option in IGUA v0.2.0, similar to MMseqs2 (`--clustering-method linclust` in IGUA v0.2.0, referred to hereafter as "IGUA linclust") (1). IGUA linclust clusters gene clusters sharing a protein in O(np) runtime, where n is the total number of gene clusters, and p the total numbers of protein representatives detected by MMseqs. Briefly, having built the protein membership matrix (**Figure 1B** of the main manuscript), rather than hierarchical clustering, the "linclust" method performs agglomerative clustering: the p MMseqs protein clusters are used to split gene clusters to be clustered into overlapping subsets. For each subset i which covers all the gene clusters containing a protein part of MMSeqs cluster i, the longest gene cluster (in number of amino acids [AA]) is selected as the representative. Every other member of the subset (at most n) is compared with normalized pairwise Manhattan distances, and merged into the same GCF when the distance is below a user-defined threshold. *The rationale is the same as the MMseqs linclust method, which uses k-mers to define the subsets.*
While obviously O(np) runtime, depending on the dataset diversity, this may converge towards O(n^2) in the case where the gene clusters do not have many proteins in common. In such a case, a linear O(n) runtime could be achieved by subsampling a fixed number of protein clusters to evaluate, rather than all p protein features.

After benchmarking IGUA linclust on our speed/memory benchmarking datasets (see section "Speed and memory benchmarking using species representative genomes" in the main manuscript), there was no increase in performance relative to the default IGUA method (which uses average linkage hierarchical clustering; **Supplementary Methods Figure SM1**).

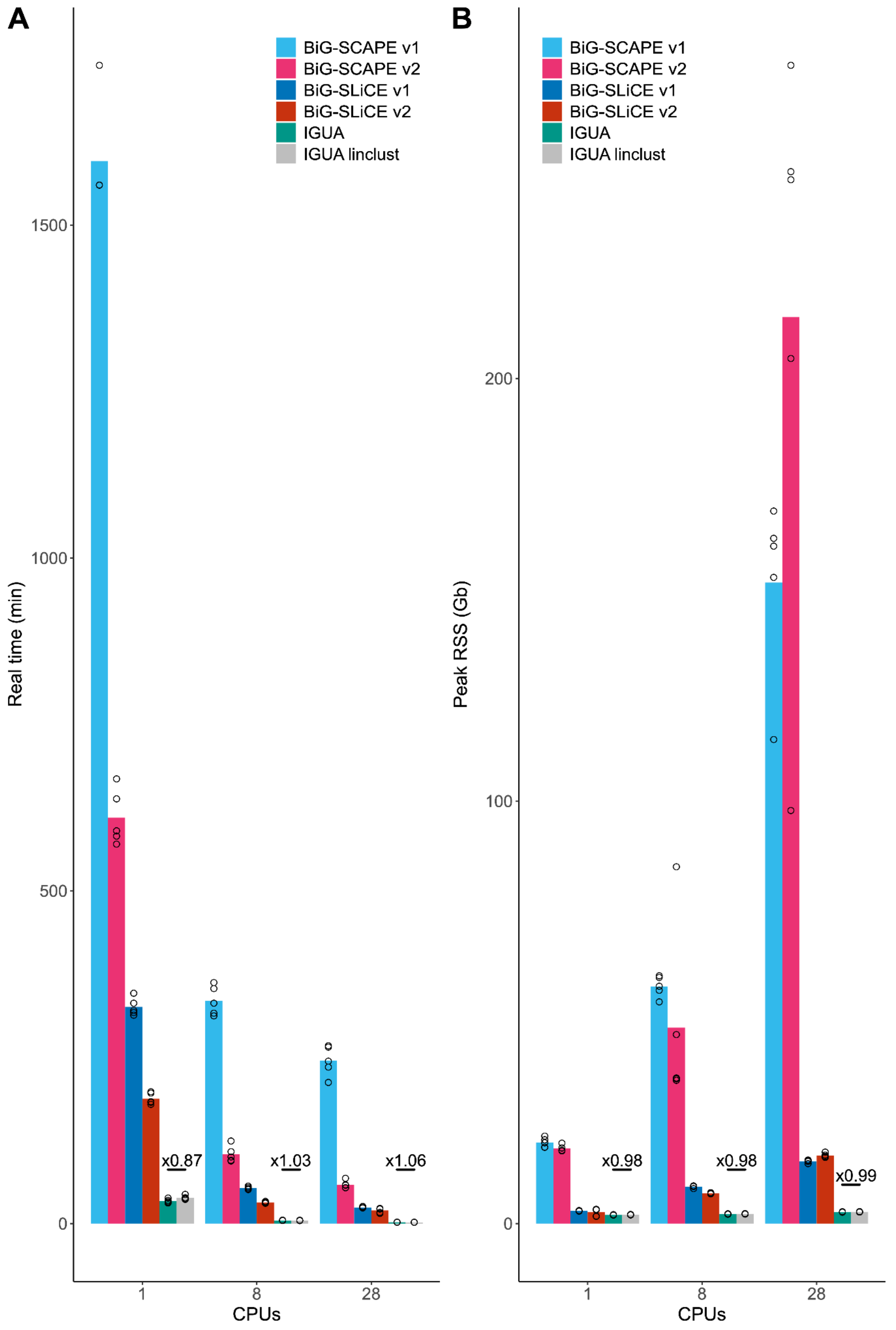

Supplementary Methods Figure SM1. (A) Average time (real/"wall clock" time, in minutes; Y-axis) and **(B)** memory usage (peak resident set size [RSS] in GB; Y-axis) for BiG-SCAPE v1 and v2, BiG-SLiCE v1 and v2, IGUA, and IGUA linclust, using 1, 8, or 28 CPUs (X-axes). For each tool, GCF delineation was carried out using antiSMASH BGCs from five diverse subsets of genomes from the proGenomes3 database (≈5,000 genomes each). Dots overlapping each bar denote raw values for each proGenomes3 subset. Text shows speed and memory of IGUA linclust (gray), relative to the default IGUA method (green).

An additional benchmark to formally assess computational complexity was performed using antiSMASH BGCs from diverse prokaryotic genomes. Briefly, antiSMASH v7.1.0 (2) was used to detect BGCs in all species representative genomes within the proGenomes v3 database ("--genefinding-tool prodigal-m" plus default parameters) (3). BGCs were then randomly sampled from this dataset to construct GenBank files with the following number of antiSMASH BGCs (in thousands): 1-10, 12.5, 15, 17.5, 20, 25, 50, 75, 100, 150, or 200. For each dataset size, random sampling was conducted two additional times (i.e., to evaluate each dataset size in triplicate). IGUA v0.2.1 was then used to cluster each dataset into GCFs using 8 CPUs and either (i) default settings (i.e., "--clustering-method average" for average linkage hierarchical clustering) or (ii) linear clustering (i.e., "--clustering-method linclust"), with all remaining parameters set to their defaults. The time it took to perform protein clustering *alone* (i.e., just the hierarchical clustering or linear clustering step within the larger IGUA pipeline) was logged and plotted in R v4.6.1 (4) using the ggplot v4.0.3 (5) and dplyr v1.2.1 (6) packages.

Overall, the computational complexity benchmark confirmed that IGUA's linclust method was indeed linear in terms of time complexity (**Supplementary Methods Figures SM2, SM3**). However, we opted to keep average linkage hierarchical clustering as the default method in IGUA, as it slightly outperformed the linear clustering method based on the metric of GCF purity (**Supplementary Figure S1**).

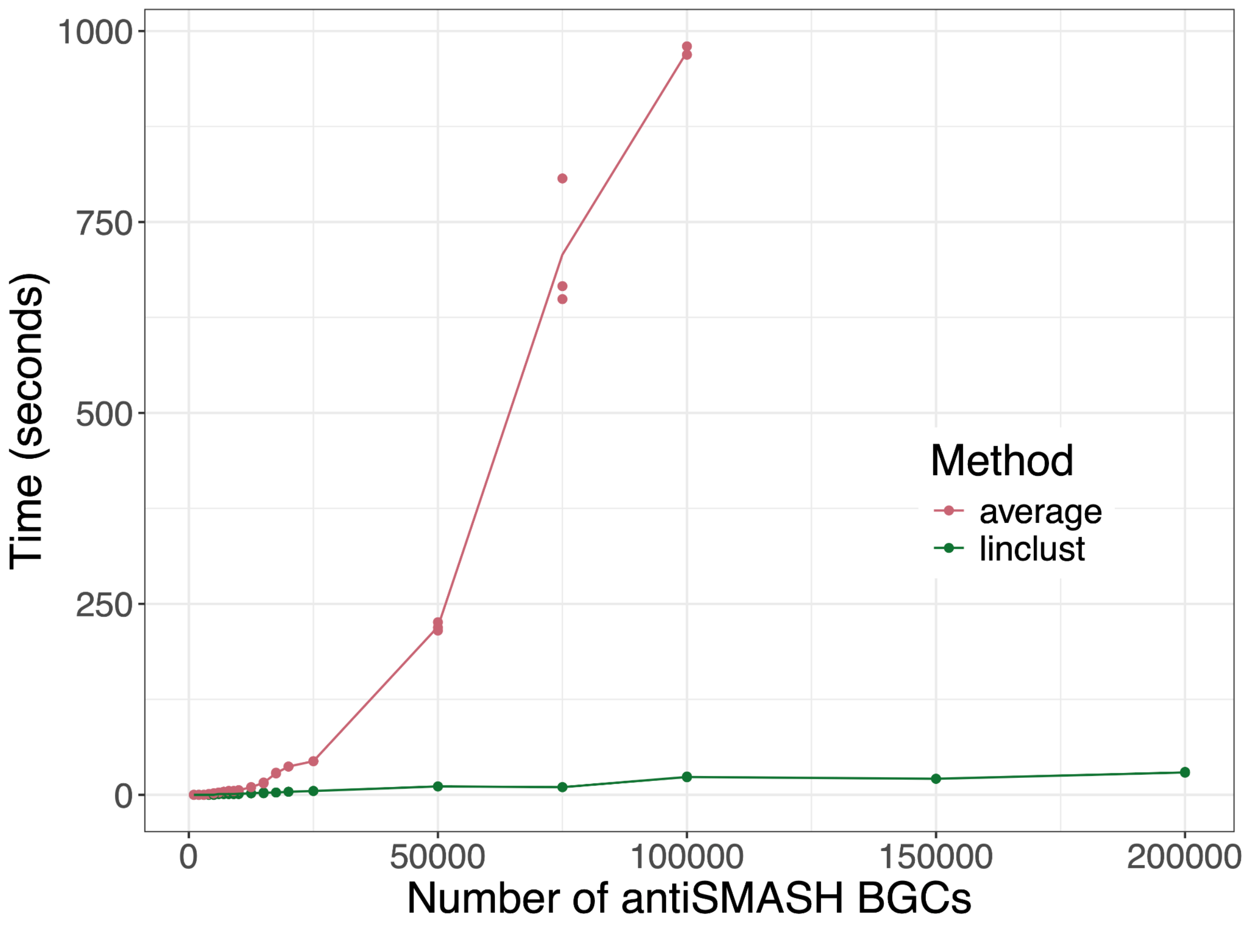

Supplementary Methods Figure SM2. Time (in seconds; Y-axis) needed for IGUA's average linkage hierarchical clustering (Method "average"; "--clustering-method average") or linear clustering (Method "linclust"; "--clustering-method linclust") step to complete on BGC datasets of varying sizes (X-axis) using 8 CPUs. GCF delineation was carried out using antiSMASH BGCs detected in species representative genomes from the proGenomes v3 database. Dots denote raw values for each replicate (3 per dataset size). For the 150,000- and 200,000-BGC datasets, average linkage hierarchical clustering could not be executed using 8 CPUs and is thus not shown.

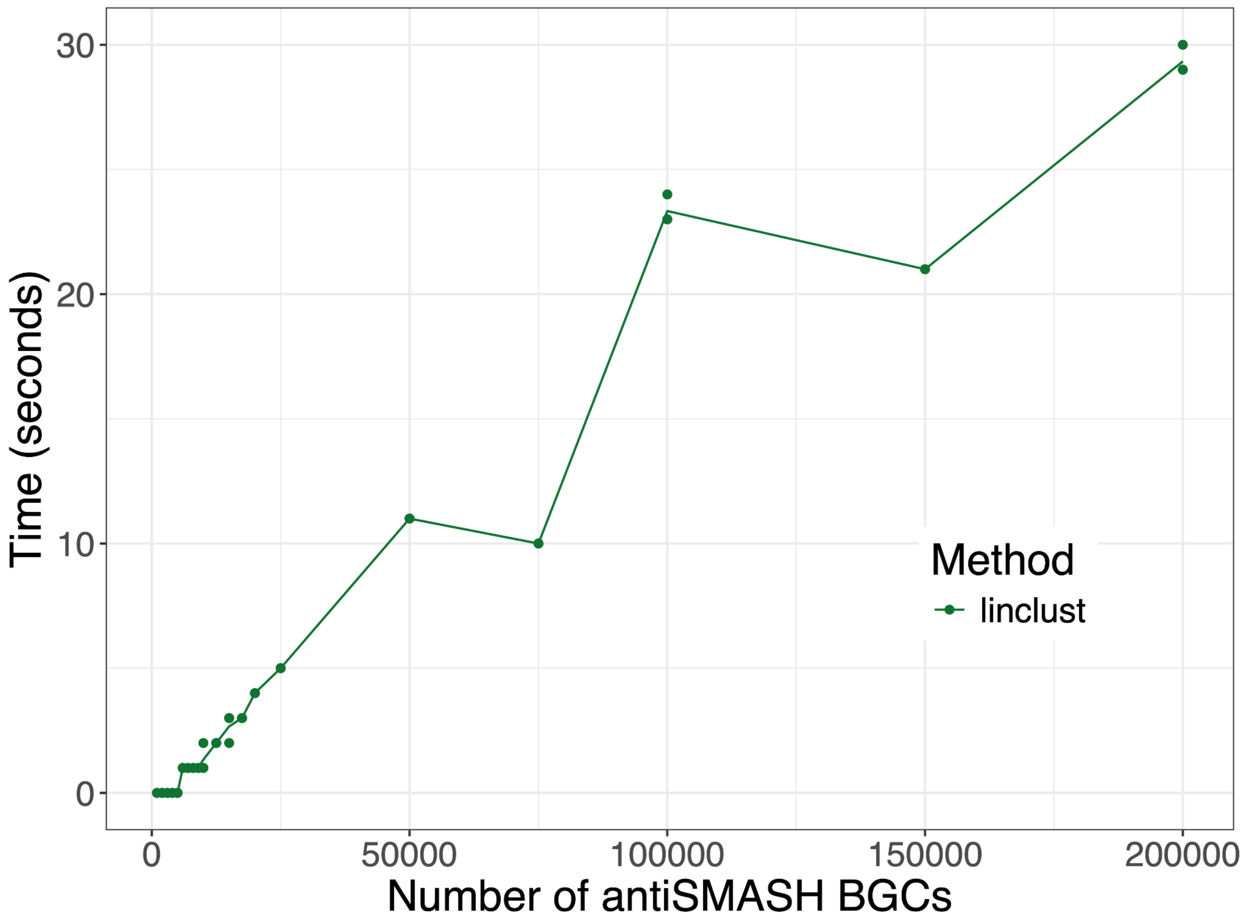

Supplementary Methods Figure SM3. Time (in seconds; Y-axis) needed for IGUA's linear clustering (Method "linclust"; "--clustering-method linclust") step to complete on BGC datasets of varying sizes (X-axis) using 8 CPUs. GCF delineation was carried out using antiSMASH BGCs detected in species representative genomes from the proGenomes v3 database. Dots denote raw values for each replicate (3 per dataset size).

Clustering of >2.8 million GECCO BGCs from >900,000 prokaryotic genomes
To showcase its speed and scalability, IGUA was used to cluster all BGCs detected in the proGenomes3 database (3) into GCFs. Briefly, GenBank records for all BGCs identified in all proGenomes3 genomes were downloaded (*n* = 2,826,569 total BGCs from 907,388 total genomes, detected using GECCO, a ML-based BGC detection

method, as described previously; https://progenomes.embl.de/data/progenomes3_gecco_clusters.gbk.gz) (3, 7). IGUA v0.1.0 was used to cluster all 2,826,569 BGCs, plus 2,502 experimentally validated BGCs from MIBiG v3.1, into GCFs (using 72 CPUs, linkage method = "average", and clustering distance = 0.80; **Supplementary Table S11**). Runtime and memory usage were logged by enabling the “report” option in the Nextflow configuration file.

To visualize proGenomes3 GCFs in two-dimensional space, a UMAP (8) was constructed using GCF representatives output by IGUA (*n* = 56,960 GCF representative BGCs). To construct the UMAP, pyHMMER v0.8.2 (9) was used to annotate each IGUA GCF representative BGC (28 CPUs and default settings), using Pfam v35.0 profile hidden Markov models (pHMMs; downloaded from the Pfam FTP site (https://ftp.ebi.ac.uk/pub/databases/Pfam/releases/Pfam35.0/Pfam-A.hmm.gz) (10). From this, a Pfam domain composition matrix was computed (via https://zenodo.org/records/21976829/files/umap_bgcs.py?download=1) and extracted for UMAP construction. The UMAP was produced in R v4.3.1, using the packages Seurat v5.0.2 (11), Signac v1.12.0 (12), reticulate v1.35.0 (https://CRAN.R-project.org/package=reticulate, accessed 23 December 2024), and clustree v0.5.1 (13). Briefly, the Pfam composition matrix was imported using the scipy.sparse$load_npz function and converted into a Seurat object (CreateSeuratObject(), assay = “peaks”) via intermediate conversions to an RDS object, followed by a chromatin assay (CreateChromatinAssay()). This was followed by normalization and selection of top features (RunTFIDF() and FindTopFeatures(), respectively, with min.cutoff =”q0” for the latter) prior to dimensional reduction with singular value decomposition (SVD) using RunSVD(). Thereafter, RunUMAP() was run on the top 30 SVD dimensions, with reduction = “lsi”, n.neighbors = 40L, and min.dist = 0.2. FindNeighbours() was used to create a nearest-neighbour graph (reduction = “lsi”, dims = 1:30). For selection of an appropriate clustering resolution, a clustree was created for resolutions 0.10-0.90 with 0.05 increments (**Supplementary Figure S5**). Finally, the clusters of the UMAP were created using FindClusters() (resolution = 0.65, algorithm = 3). The resulting Seurat object was saved as pfams_proG3.RDS using saveRDS(). The predicted biosynthetic class(es) assigned to each BGC (obtained using GECCO as described previously) (3) were extracted from each BGC’s GenBank record. When >1 protocluster/biosyn_class object was present, the BGC was labelled as “Mixed”. The predicted biosynthetic class(es) assigned to the majority of BGCs in each GCF was attached to the pfams_proG3.RDS object using AddMetaData() (**Supplementary Figure S5**, **Supplementary Table S12**).

Clustering of secretion system clusters

For secretion system clustering, metadata for all secretion systems in TXSSdb (14) (i.e., nucleotide accession numbers, start/end coordinates, and secretion system type classifications) were downloaded (*n* = 11,939 total secretion system entries; http://macsydb.web.pasteur.fr/txssdb/_design/txssdb/index.html, accessed 27 September 2024). Secretion systems classified by TXSSdb as “single_locus” secretion systems were used in subsequent steps (*n* = 10,784 TXSSdb single-locus secretion

systems). The “entrez_fetch” command in the rentrez package (v1.2.3) (15) in R v4.3.2 was used to query NCBI’s Nucleotide database (16) for each single-locus secretion system, using NCBI Nucleotide accession numbers and secretion system start/end coordinates provided by TXSSdb. This resulted in a total of 10,576 valid GenBank files (one per single-locus secretion system), which were concatenated into a single GenBank file using BioPython v1.84 (17). IGUA v0.1.0 was then used to cluster all 10,576 TXSSdb single-locus secretion systems into GCFs (linkage method = "average", clustering distance = 0.80; **Supplementary Table S13**). The “entrez_search” and “entrez_summary” commands in rentrez were further used to download the host bacterium genome for each secretion system, and each host bacterium genome underwent taxonomic classification using GTDB release214/GTDB-Tk v2.3.2 (i.e., via GTDB-Tk’s “classify_wf” workflow with “--cpus 28” and default settings; **Supplementary Table S14**) (18, 19).

A UMAP of the IGUA GCF representative secretion systems was constructed as described above (see section “Clustering of >2.8 million GECCO BGCs from >900,000 prokaryotic genomes”), with the following alterations: the protein domain composition matrix was computed using https://zenodo.org/records/21976829/files/umap_secretion.py?download=1, the RunUMAP() options min.dist and n.neighbours were not changed from default settings, and the clustering resolution for FindClusters() was set to 0.35. The Seurat object was saved as pfams_secretion.RDS, and the attached metadata consisted of (a) secretion system types (provided by TXSSdb), and (b) host bacterium taxonomic classifications (per GTDB-Tk; **Supplementary Figure S6**, **Supplementary Tables S14-S15**). This process was repeated a second time, but with the clustering resolution for FindClusters() set to 0.80 (the default; **Figure 4**).

Clustering of prophage clusters

For prophage clustering, all prophage sequences in Prophage-DB (20) were downloaded (i.e., bacterial_host_prophages.tar.gz, archaeal_host_prophages.tar.gz, unknown_host_prophages.tar.gz, and all corresponding metadata; https://doi.org/10.5061/dryad.3n5tb2rs5, accessed 26 November 2024). Prokka v1.14.6 (21) was used to annotate each prophage using its respective FASTA file as input, the “Viruses” database (“--kingdom Viruses”), and the “--addgenes” and “--compliant” options enabled (remaining options were set to their defaults). Annotated GenBank files produced by Prokka (*n* = 356,776 files, one per Prophage-DB prophage) were concatenated into a single GenBank file and clustered into GCFs using IGUA (linkage method = "average", clustering distance = 0.80; **Supplementary Table S16**). Due to the large number of resulting GCFs (*n* = 213,699), IGUA was run a second time, using average linkage and a higher clustering distance threshold (i.e., 0.99, resulting in 106,749 GCFs; **Supplementary Table S17**). IGUA GCF representatives identified using this higher threshold (0.99) were visualized via a UMAP, which was constructed as described above, with the following changes: the protein domain composition matrix was computed using https://zenodo.org/records/21976829/files/umap_prophage.py?download=1, and the options min.dist and n.neighbours for RunUMAP() and clustering resolution for

FindClusters() were not altered from default settings. The SeuratObject was saved as pfams_prophage.RDS, and the attached metadata consisted of host bacterium taxonomic classifications (provided by Prophage-DB; **Supplementary Figure S7**, **Supplementary Table S18**).

Clustering of genomic islands

For genomic island clustering, each individual IslandViewer 4 dataset (i.e., genomic islands detected using one of four different methods: IslandPick, IslandPath-DIMOB, SIGI-HMM, or Islander) was downloaded as a tab-separated (TSV) file (https://www.pathogenomics.sfu.ca/islandviewer/download/datasets/all_gis_islandpick_iv4.txt.tar.gz, https://www.pathogenomics.sfu.ca/islandviewer/download/datasets/all_gis_islandpath_dimob_iv4.txt.tar.gz, https://www.pathogenomics.sfu.ca/islandviewer/download/datasets/all_gis_sigi_hmm_iv4.txt.tar.gz, https://www.pathogenomics.sfu.ca/islandviewer/download/datasets/all_gis_islander_iv4.txt.tar.gz, respectively, downloaded 8 November 2025) (22–26). The “entrez_fetch” command in the rentrez package (v1.2.3) (15) in R v4.3.2 was used to query NCBI’s Nucleotide database (16) for each genomic island, using NCBI Nucleotide accession numbers and genomic island start/end coordinates provided by IslandViewer 4. This resulted in a total of 902,158 valid GenBank files (one per genomic island), which were concatenated into a single GenBank file using BioPython v1.84 (17). IGUA v0.1.0 was then used to cluster all 902,158 genomic islands into GCFs (linkage method = "average", clustering distance = 0.80; **Supplementary Table S19**).

IGUA representative GCFs were visualized via a UMAP, constructed as described above with the following alterations: the protein domain composition matrix was computed using https://zenodo.org/records/21976829/files/umap_genomic_islands.py?download=1, and the options min.dist and n.neighbours for RunUMAP() and clustering resolution for FindClusters() were not altered from default settings. The SeuratObject was saved as pfams_genomic_islands.RDS, and the attached metadata consisted of genomic island detection method information (**Supplementary Figures S8-S9**, **Supplementary Table S20**).

Clustering of phage-plasmids

For phage-plasmid clustering, Supplementary Dataset S1 from Pfeifer and Rocha (27), which contains NCBI Nucleotide accession numbers for a selection of phage, plasmids, and phage-plasmids, was downloaded (accessed 12 December 2025). The “entrez_fetch” command in the rentrez package (v1.2.3) (15) in R v4.3.2 was used to query NCBI’s Nucleotide database (16) for each phage/plasmid/phage-plasmid, using the NCBI Nucleotide accession number for each. This resulted in a total of 25,197 valid GenBank files (one per each individual phage/plasmid/phage-plasmid), which were concatenated into a single GenBank file using BioPython v1.84 (17). IGUA v0.1.0 was then used to cluster all 25,197 phage/plasmids/phage-plasmids into GCFs (linkage method = "average", clustering distance = 0.80; **Supplementary Table S21**).

IGUA representative GCFs were visualized via a UMAP, constructed as described above with the following changes: the protein domain composition matrix was computed using https://zenodo.org/records/21976829/files/umap_phage_plasmid.py?download=1, and the options min.dist and n.neighbours for RunUMAP() were set to 0.4 and 30L, respectively. The clustering resolution for FindClusters() was set to 0.6, based on a clustree visualization (**Supplementary Figure S10**). The SeuratObject was saved as pfams_phage_plasmid.RDS, and the attached metadata consisted of classes (i.e., "Phage", "Plasmid", and "Phage-Plasmid [P-P]"; **Supplementary Figures S9-S10**, **Supplementary Table S22).**

Clustering of CAZyme gene clusters

To construct a large dataset of CAZyme (carbohydrate active enzyme) gene clusters, the latest version of MGnify's "human-gut" database (v2.0.2) was downloaded via the MGnify FTP site (https://ftp.ebi.ac.uk/pub/databases/metagenomics/mgnify_genomes/human-gut/v2.0.2/), resulting in a set of 289,232 microbial genomes and metagenome-assembled genomes (MAGs) with associated metadata (https://ftp.ebi.ac.uk/pub/databases/metagenomics/mgnify_genomes/human-gut/v2.0.2/genomes-all_metadata.tsv) (28). Each GFF file was decompressed using gunzip and its FASTA component (containing the [meta]genome sequence) was stored as a separate FASTA file (cat $f | sed -n '/^##FASTA/,$p' | grep -v "##FASTA" >> ${f%.gff}.fna). The FASTA file for each genome/MAG (*n* = 289,232) was then supplied as input to run_dbCAN v5.2.1 (29), which was used to detect CAZyme gene clusters using the easy_CGC workflow and the following parameters: --mode meta (to specify that metagenomic contigs were supplied as input); --cgc (to enable CGC-related databases); --db_dir (to specify the path to the latest dbCAN database, downloaded on 12 December 2025 using the "run_dbcan database" command) (29). The resulting genome/MAG GFF file, plus the CAZyme gene cluster coordinate file (both produced by run_dbCAN) were then supplied as input to gff2gbk v0.1.0 (https://github.com/lmc297/gff2gbk; -i and -r, respectively), which was used to extract the CAZyme gene cluster from the GFF file and convert it to a GenBank file using the following parameters: --contig "Contig ID" (the contig ID column name in the CAZyme gene cluster coordinate file); --start "Cluster Start" --end "Cluster End" (the gene cluster start and end coordinate column names in the CAZyme gene cluster coordinate file); --add_translations (to add translations to CDS features); --igua (to output a GenBank file that is compatible with IGUA).

Overall, the above approach resulted in a total of 9,010,369 valid GenBank files (one per CAZyme gene cluster); these GenBank files were concatenated into a single GenBank file alongside the 579 experimentally characterized CAZyme gene clusters from the dbCAN-PUL database (30) with valid GenBank files (https://pro.unl.edu/static/DBCAN-PUL/, accessed 8 November 2025) using BioPython v1.84 (17). IGUA v0.1.0 was then used to cluster all 9,010,948 CAZyme gene clusters into GCFs (linkage method = "average", clustering distance = 0.80; **Supplementary Table S23**).

IGUA representative GCFs were visualized via a UMAP, constructed as described previously with the following alterations: the protein composition matrix was computed using https://zenodo.org/records/21976829/files/umap_CGCs.py?download=1, and the options min.dist and n.neighbours for RunUMAP() and clustering resolution for FindClusters() were not altered from default settings. The SeuratObject was saved as pfams_CGCs.RDS, and the attached metadata consisted of taxonomic information and isolation location from MGnify (**Supplementary Figures S9** and **S11**, **Supplementary Table S24**).

Supplementary References

1. Steinegger,M. and Söding,J. (2017) MMseqs2 enables sensitive protein sequence searching for the analysis of massive data sets. *Nat. Biotechnol.*, **35**, 1026–1028.

2. Blin,K., Shaw,S., Augustijn,H.E., Reitz,Z.L., Biermann,F., Alanjary,M., Fetter,A., Terlouw,B.R., Metcalf,W.W., Helfrich,E.J.N., *et al.* (2023) antiSMASH 7.0: new and improved predictions for detection, regulation, chemical structures and visualisation. *Nucleic Acids Res.*, **51**, W46–W50.

3. Fullam,A., Letunic,I., Schmidt,T.S.B., Ducarmon,Q.R., Karcher,N., Khedkar,S., Kuhn,M., Larralde,M., Maistrenko,O.M., Malfertheiner,L., *et al.* (2022) proGenomes3: approaching one million accurately and consistently annotated high-quality prokaryotic genomes. *Nucleic Acids Res.*, **51**, D760–D766.

4. R Core Team (2026) R: A language and environment for statistical computing. 10.32614/r.manuals.

5. Wickham,H. ggplot2: Elegant Graphics for Data Analysis. Springer-Verlag New York, 2016.

6. Wickham,H., François,R., Henry,L., Müller,K. and Vaughan,D. (2014) dplyr: A Grammar of Data Manipulation. *CRAN: Contributed Packages*, 10.32614/cran.package.dplyr.

7. Carroll,L.M., Larralde,M., Fleck,J.S., Ponnudurai,R., Milanese,A., Cappio,E. and Zeller,G. (2021) Accurate *de novo* identification of biosynthetic gene clusters with GECCO. *bioRxiv*, 10.1101/2021.05.03.442509.

8. McInnes,L., Healy,J., Saul,N. and Großberger,L. (2018) UMAP: Uniform Manifold Approximation and Projection. *Journal of Open Source Software*, **3**, 861.

9. Larralde,M. and Zeller,G. (2023) PyHMMER: a Python library binding to HMMER for efficient sequence analysis. *Bioinformatics*, **39**.

10. Mistry,J., Chuguransky,S., Williams,L., Qureshi,M., Salazar,G.A., Sonnhammer,E.L.L., Tosatto,S.C.E., Paladin,L., Raj,S., Richardson,L.J., *et al.* (2021) Pfam: The protein families database in 2021. *Nucleic Acids Res*, **49**, D412–D419.

11. Hao,Y., Stuart,T., Kowalski,M.H., Choudhary,S., Hoffman,P., Hartman,A., Srivastava,A., Molla,G., Madad,S., Fernandez-Granda,C., *et al.* (2024) Dictionary learning for integrative, multimodal and scalable single-cell analysis. *Nat Biotechnol*, **42**, 293–304.

12. Stuart,T., Srivastava,A., Madad,S., Lareau,C.A. and Satija,R. (2021) Single-cell chromatin state analysis with Signac. *Nat Methods*, **18**, 1333–1341.

13. Zappia,L. and Oshlack,A. (2018) Clustering trees: a visualization for evaluating clusterings at multiple resolutions. *Gigascience*, **7**.

14. Abby,S.S., Cury,J., Guglielmini,J., Néron,B., Touchon,M. and Rocha,E.P.C. (2016) Identification of protein secretion systems in bacterial genomes. *Scientific Reports*, **6**, 1–14.

15. Winter,D.J. (2017) rentrez: An R package for the NCBI eUtils API. *The R Journal*, **9**, 520–526.

16. Sayers,E.W., Bolton,E.E., Brister,J.R., Canese,K., Chan,J., Comeau,D.C., Connor,R., Funk,K., Kelly,C., Kim,S., *et al.* (2021) Database resources of the national center for biotechnology information. *Nucleic Acids Res*, **50**, D20–D26.

17. Cock,P.J.A., Antao,T., Chang,J.T., Chapman,B.A., Cox,C.J., Dalke,A., Friedberg,I., Hamelryck,T., Kauff,F., Wilczynski,B., *et al.* (2009) Biopython: freely available Python tools for computational molecular biology and bioinformatics. *Bioinformatics*, **25**, 1422–1423.

18. Parks,D.H., Chuvochina,M., Rinke,C., Mussig,A.J., Chaumeil,P.-A. and Hugenholtz,P. (2021) GTDB: an ongoing census of bacterial and archaeal diversity through a phylogenetically consistent, rank normalized and complete genome-based taxonomy. *Nucleic Acids Res.*, **50**, D785–D794.

19. Chaumeil,P.-A., Mussig,A.J., Hugenholtz,P. and Parks,D.H. (2022) GTDB-Tk v2: memory friendly classification with the genome taxonomy database. *Bioinformatics*, **38**, 5315–5316.

20. Dieppa-Colón,E., Martin,C. and Anantharaman,K. (2024) Prophage-DB: A comprehensive database to explore diversity, distribution, and ecology of prophages. *bioRxiv*, 10.1101/2024.07.11.603044.

21. Seemann,T. (2014) Prokka: rapid prokaryotic genome annotation. *Bioinformatics*, **30**, 2068–2069.

22. Bertelli,C., Laird,M.R., Williams,K.P., Simon Fraser University Research Computing Group, Lau,B.Y., Hoad,G., Winsor,G.L. and Brinkman,F.S.L. (2017) IslandViewer 4: expanded prediction of genomic islands for larger-scale datasets. *Nucleic Acids Res*, **45**, W30–W35.

23. Langille,M.G.I., Hsiao,W.W.L. and Brinkman,F.S.L. (2008) Evaluation of genomic island predictors using a comparative genomics approach. *BMC Bioinformatics*, **9**, 329.

24. Hsiao,W., Wan,I., Jones,S.J. and Brinkman,F.S.L. (2003) IslandPath: aiding detection of genomic islands in prokaryotes. *Bioinformatics*, **19**, 418–420.

25. Waack,S., Keller,O., Asper,R., Brodag,T., Damm,C., Fricke,W.F., Surovcik,K.,

Meinicke,P. and Merkl,R. (2006) Score-based prediction of genomic islands in prokaryotic genomes using hidden Markov models. *BMC Bioinformatics*, **7**, 142.

26. Hudson,C.M., Lau,B.Y. and Williams,K.P. (2015) Islander: a database of precisely mapped genomic islands in tRNA and tmRNA genes. *Nucleic Acids Res.*, **43**, D48–53.

27. Pfeifer,E. and Rocha,E.P.C. (2024) Phage-plasmids promote recombination and emergence of phages and plasmids. *Nat. Commun.*, **15**, 1545.

28. Richardson,L., Allen,B., Baldi,G., Beracochea,M., Bileschi,M.L., Burdett,T., Burgin,J., Caballero-Pérez,J., Cochrane,G., Colwell,L.J., *et al.* (2023) MGnify: the microbiome sequence data analysis resource in 2023. *Nucleic Acids Res.*, **51**, D753–D759.

29. Zheng,J., Ge,Q., Yan,Y., Zhang,X., Huang,L. and Yin,Y. (2023) dbCAN3: automated carbohydrate-active enzyme and substrate annotation. *Nucleic Acids Res.*, **51**, W115–W121.

30. Ausland,C., Zheng,J., Yi,H., Yang,B., Li,T., Feng,X., Zheng,B. and Yin,Y. (2021) dbCAN-PUL: a database of experimentally characterized CAZyme gene clusters and their substrates. *Nucleic Acids Res.*, **49**, D523–D528.
