# Supplementary material for: Fast, flexible gene cluster family delineation with IGUA
Source: Nucleic Acids Res. 2026 Oct 13;54(19):gkag975. doi: 10.1093/nar/gkag975 (PMC13659240; doi:10.1093/nar/gkag975)
Supplement: Supplementary file 1 [file gkag975_supplemental_files.zip › Supplementary_Figure_S10_clustree_phage_plasmid.pdf]

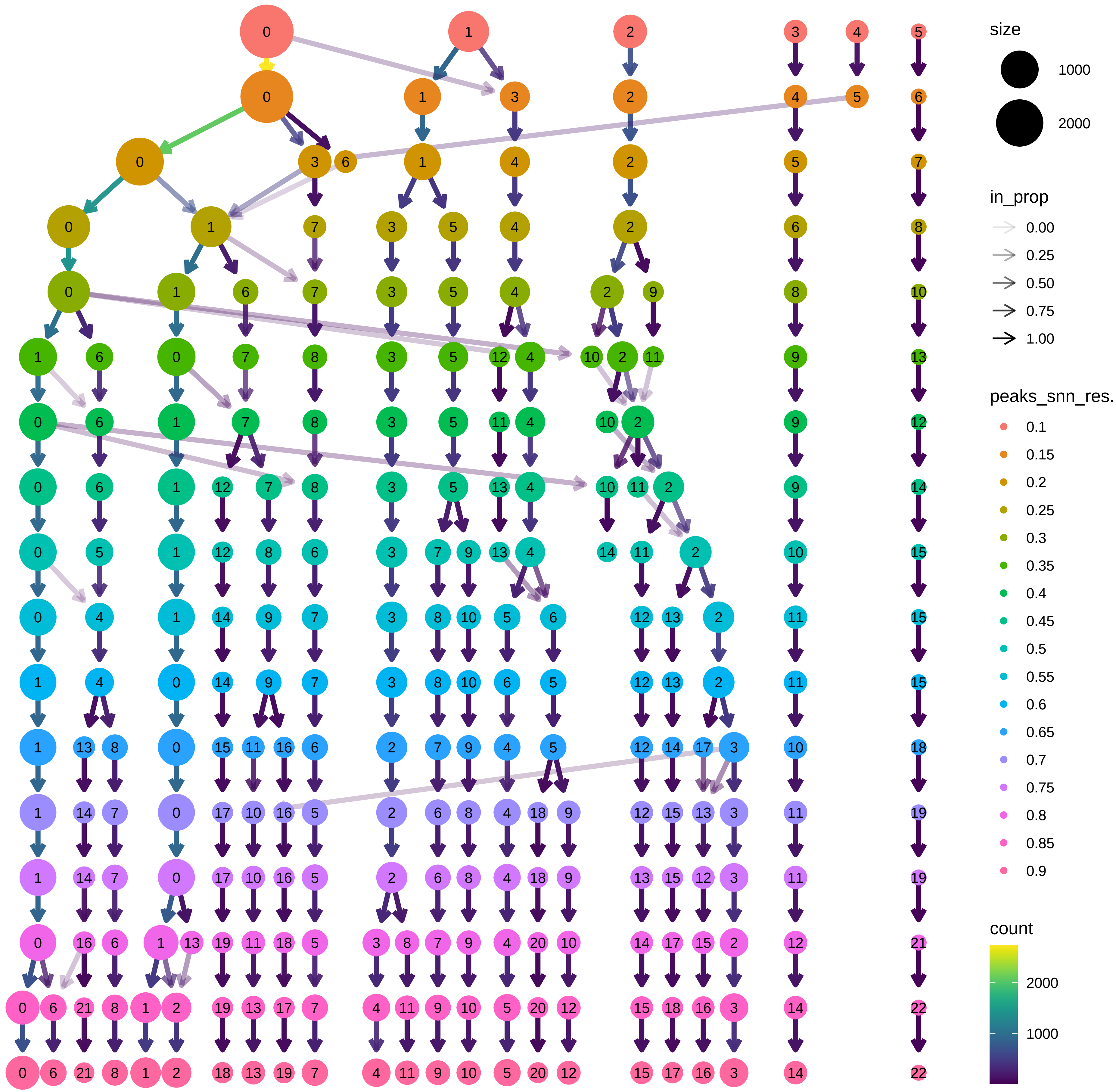

Supplementary Figure S10. Clustering tree for the phage-plasmid UMAP. “size” = the number of gene clusters in a particular cluster; “count” = the number of gene clusters carried over from a parent to child cluster; “in_prop” = the proportion of gene clusters carried over from a parent to a child cluster; “peaks_snn_res.” = the clustering resolution (as set in Seurat FindCluster()).
