# Supplementary material for: Fast, flexible gene cluster family delineation with IGUA
Source: Nucleic Acids Res. 2026 Oct 13;54(19):gkag975. doi: 10.1093/nar/gkag975 (PMC13659240; doi:10.1093/nar/gkag975)
Supplement: Supplementary file 1 [file gkag975_supplemental_files.zip › Supplementary_Figure_S1_w_linclust.pdf]

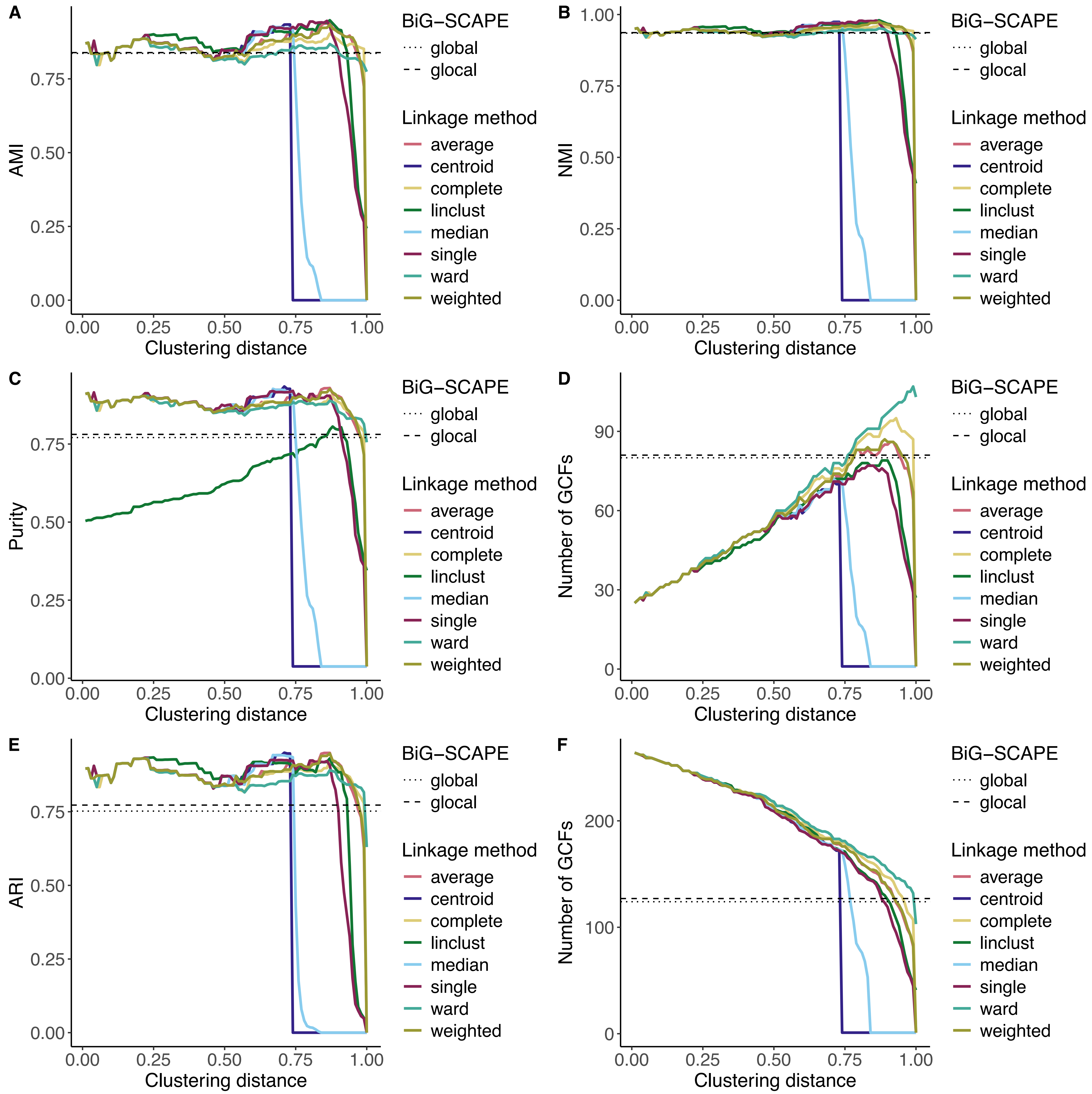

Supplementary Figure S1. (A) Adjusted mutual information (AMI), (B) normalized mutual information (NMI), (C) purity, (D) number of Gene Cluster Families (GCFs; y-axis) produced by IGUA (excluding singleton GCFs), (E) Adjusted Rand Index (ARI), and (F) number of GCFs produced by IGUA (including singleton GCFs) relative to manually curated, "ground truth" GCFs for each tested parameter combination (clustering distance, x-axis; hierarchical clustering linkage method, line color). Linclust has been included as a linkage method. Horizontal dashed lines denote values achieved by BiG-SCAPE (v1) in "global" and "glocal" mode. A clustering distance of 0.80 and the "average" linkage method were selected as IGUA's default parameters.
