# Supplementary material for: Fast, flexible gene cluster family delineation with IGUA
Source: Nucleic Acids Res. 2026 Oct 13;54(19):gkag975. doi: 10.1093/nar/gkag975 (PMC13659240; doi:10.1093/nar/gkag975)
Supplement: Supplementary file 1 [file gkag975_supplemental_files.zip › Supplementary_Figure_S2_heatmap_manually_curated_dataset_v2.pdf]

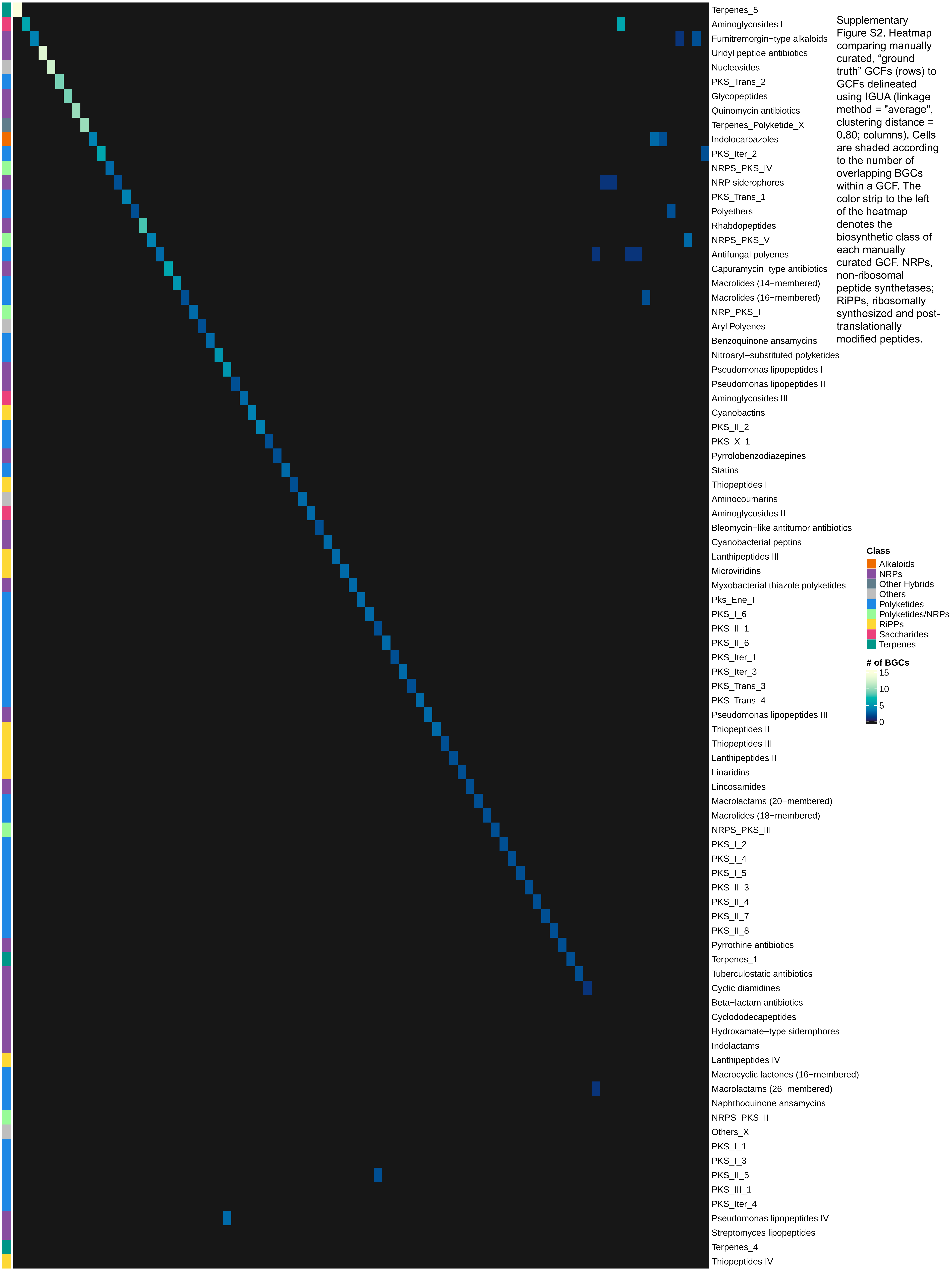


Supplementary Figure S2. Heatmap comparing manually curated, “ground truth” GCFs (rows) to GCFs delineated using IGUA (linkage method = "average", clustering distance = 0.80; columns). Cells are shaded according to the number of overlapping BGCs within a GCF. The color strip to the left of the heatmap denotes the biosynthetic class of each manually curated GCF. NRPs, non-ribosomal peptide synthetases; RiPPs, ribosomally synthesized and post-translationally modified peptides.
