# Supplementary material for: Fast, flexible gene cluster family delineation with IGUA
Source: Nucleic Acids Res. 2026 Oct 13;54(19):gkag975. doi: 10.1093/nar/gkag975 (PMC13659240; doi:10.1093/nar/gkag975)
Supplement: Supplementary file 1 [file gkag975_supplemental_files.zip › Supplementary_Figure_S3_v3.pdf]

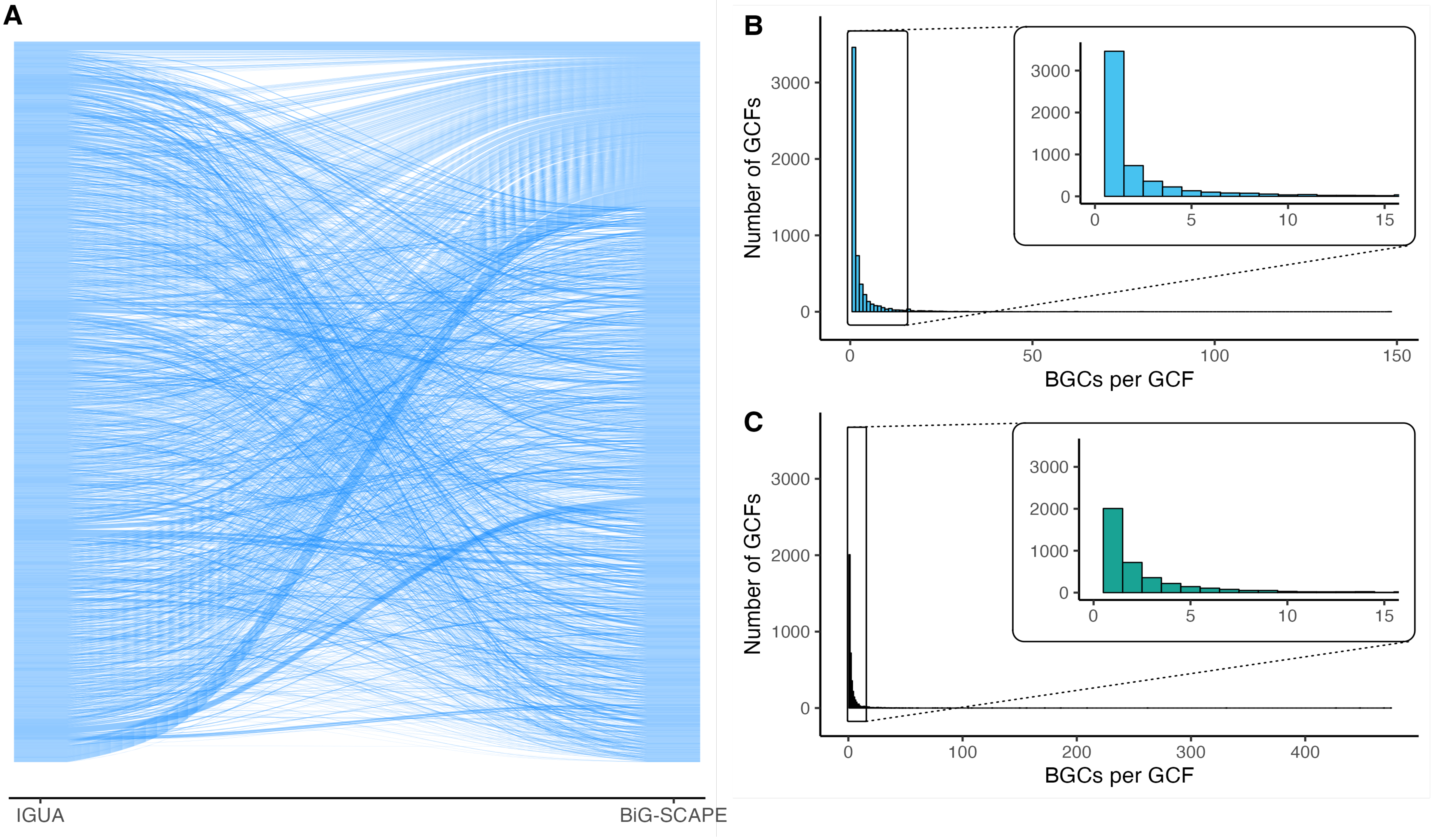

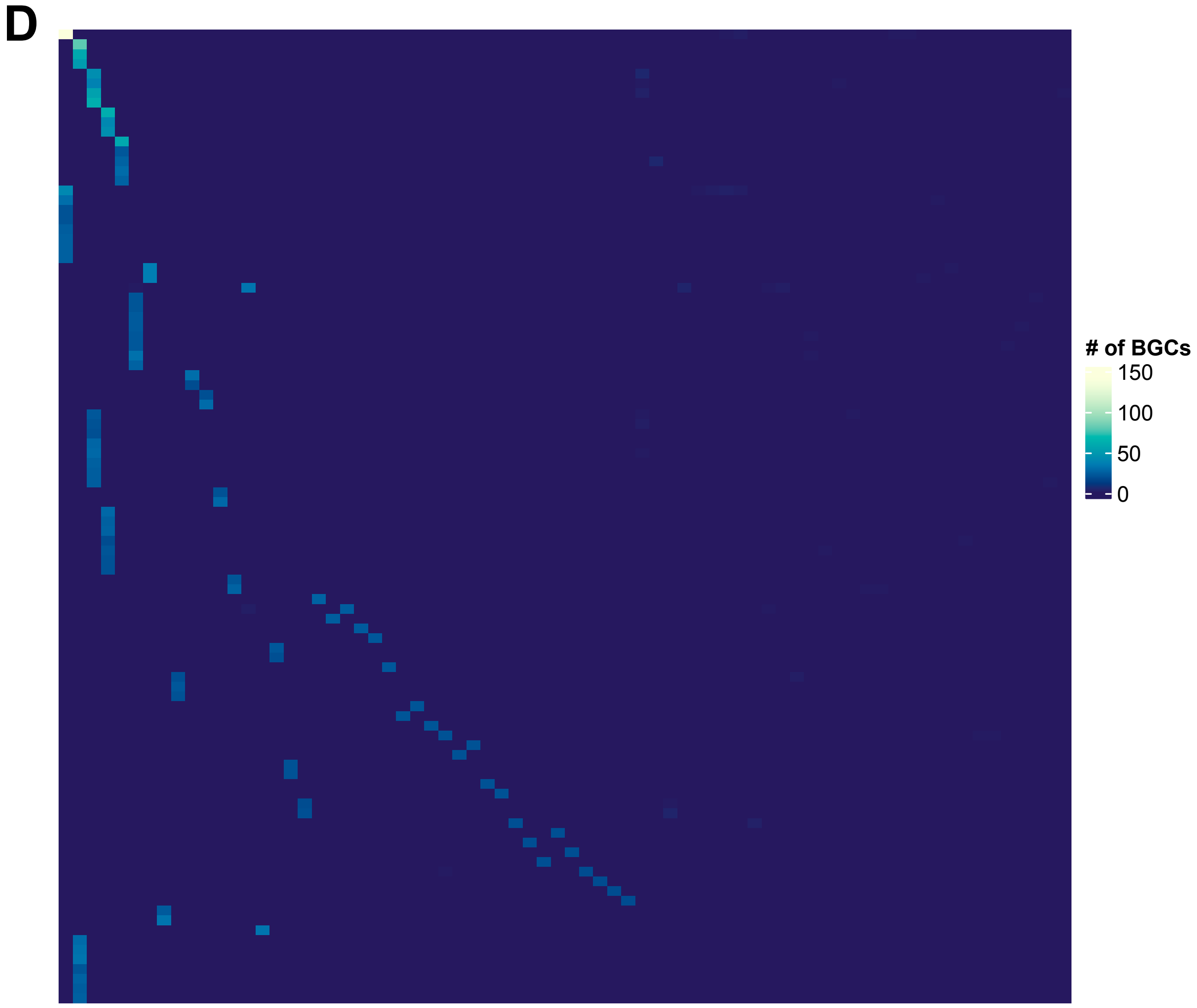

**Metrics**	With singletons	Without singletons
**AMI**	0.533	0.612
**NMI**	0.826	0.823
**ARI**	0.199	0.211

Supplementary Figure S3. Comparison of IGUA and BiG-SCAPE (v1.1.5) GCFs on a *Streptomyces* dataset, with singleton GCFs excluded. (A) Alluvial plot comparing the composition of *Streptomyces* GCFs delineated by IGUA (left) and BiG-SCAPE (right). (B and C) Histogram of the number of BGCs per *Streptomyces* GCF (X-axis) detected using BiG-SCAPE (blue, top) and IGUA (green, bottom). (D) Heatmap comparing GCFs delineated using BiG-SCAPE (rows) and IGUA (columns). For readability, only the 100 largest overlapping GCFs are shown. Metrics to the right of the heatmap pertain to the entire *Streptomyces* dataset. "AMI" = Adjusted Mutual Information; "ARI" = Adjusted Rand Index; "NMI" = Normalized Mutual Information.
