# Supplementary material for: Fast, flexible gene cluster family delineation with IGUA
Source: Nucleic Acids Res. 2026 Oct 13;54(19):gkag975. doi: 10.1093/nar/gkag975 (PMC13659240; doi:10.1093/nar/gkag975)

A

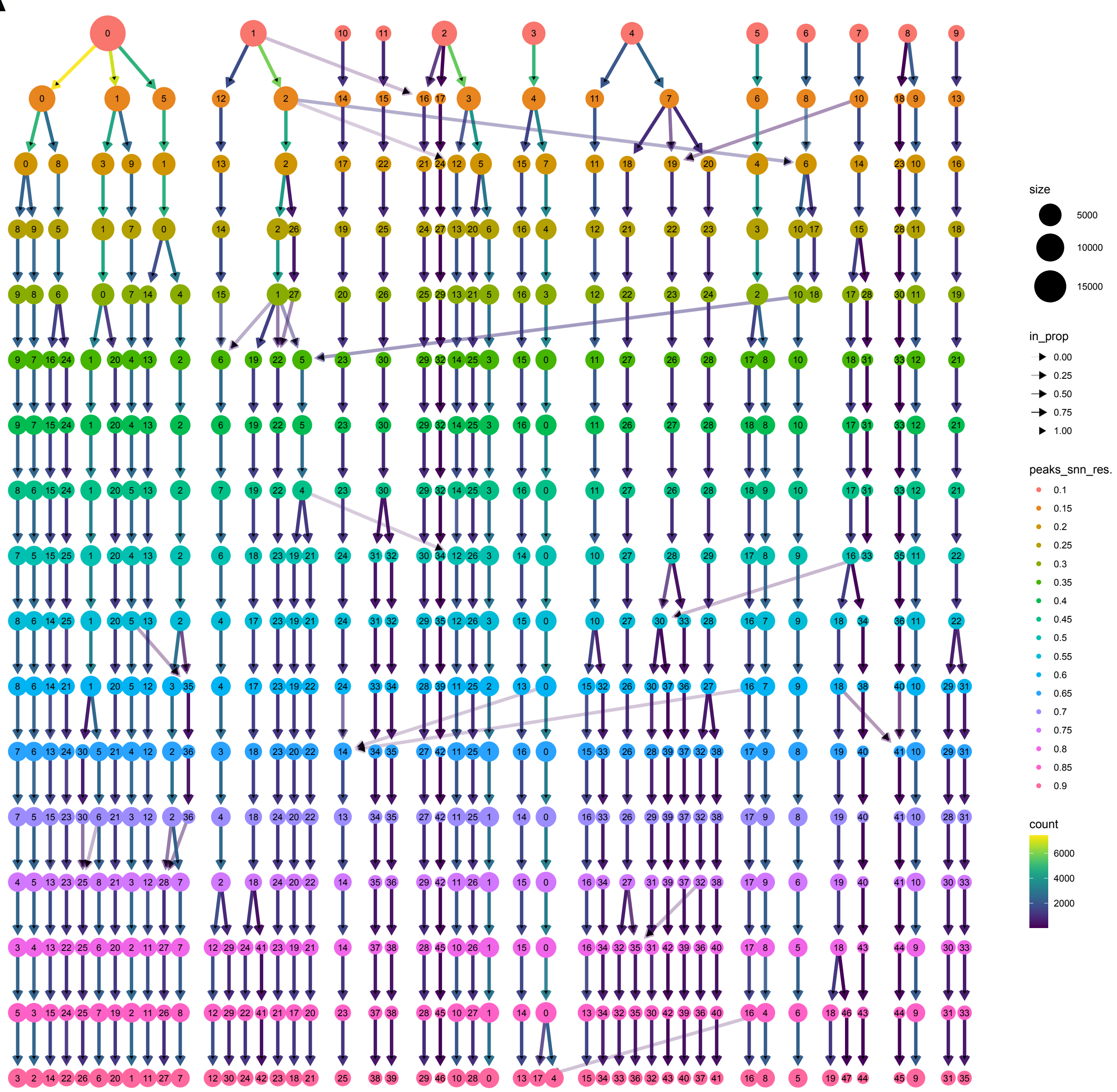

Supplementary Figure S5. (A) Clustering tree for the proGenomes3 UMAP. “size” = the number of BGCs in a particular cluster; “count” = the number of BGCs carried over from a parent to child cluster; “in_prop” = the proportion of BGCs carried over from a parent to a child cluster; “peaks_snn_res.” = the clustering resolution (as set in Seurat’s FindCluster() function). (B) proGenomes3 UMAP, colored by predicted biosynthetic class of the majority of BGCs in the GCF. Each dot represents a Gene Cluster Family (GCF) representative identified using IGUA.

B

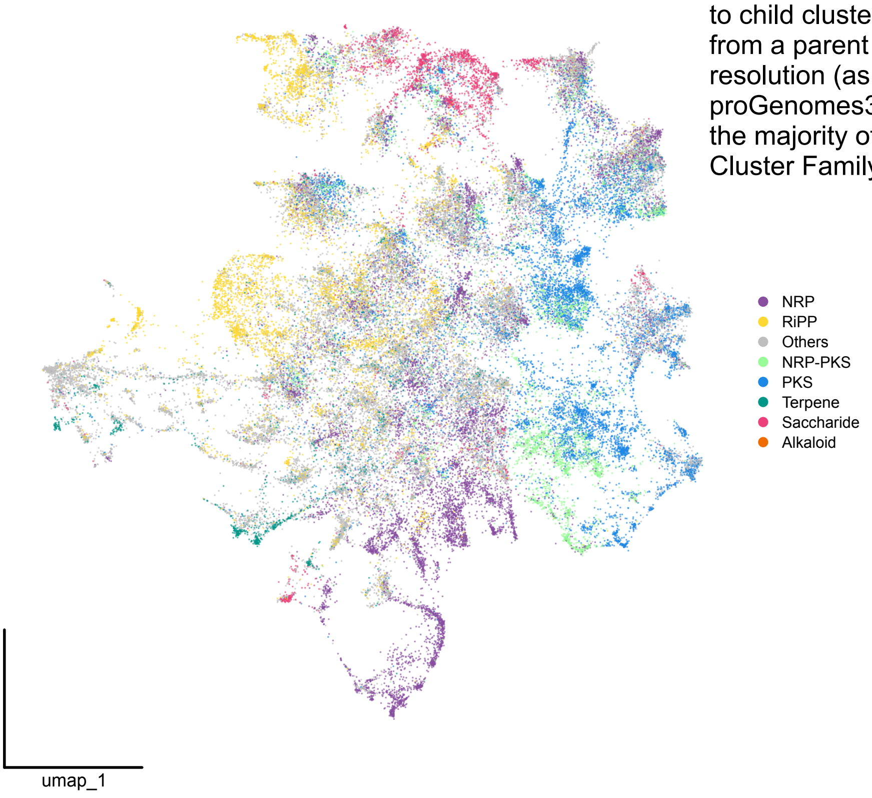

Supplement: Supplementary file 1 [file gkag975_supplemental_files.zip › Supplementary_Figure_S5_v2.pdf]
