# Supplementary material for: Fast, flexible gene cluster family delineation with IGUA
Source: Nucleic Acids Res. 2026 Oct 13;54(19):gkag975. doi: 10.1093/nar/gkag975 (PMC13659240; doi:10.1093/nar/gkag975)
Supplement: Supplementary file 1 [file gkag975_supplemental_files.zip › Supplementary_Figure_S7_v3.pdf]

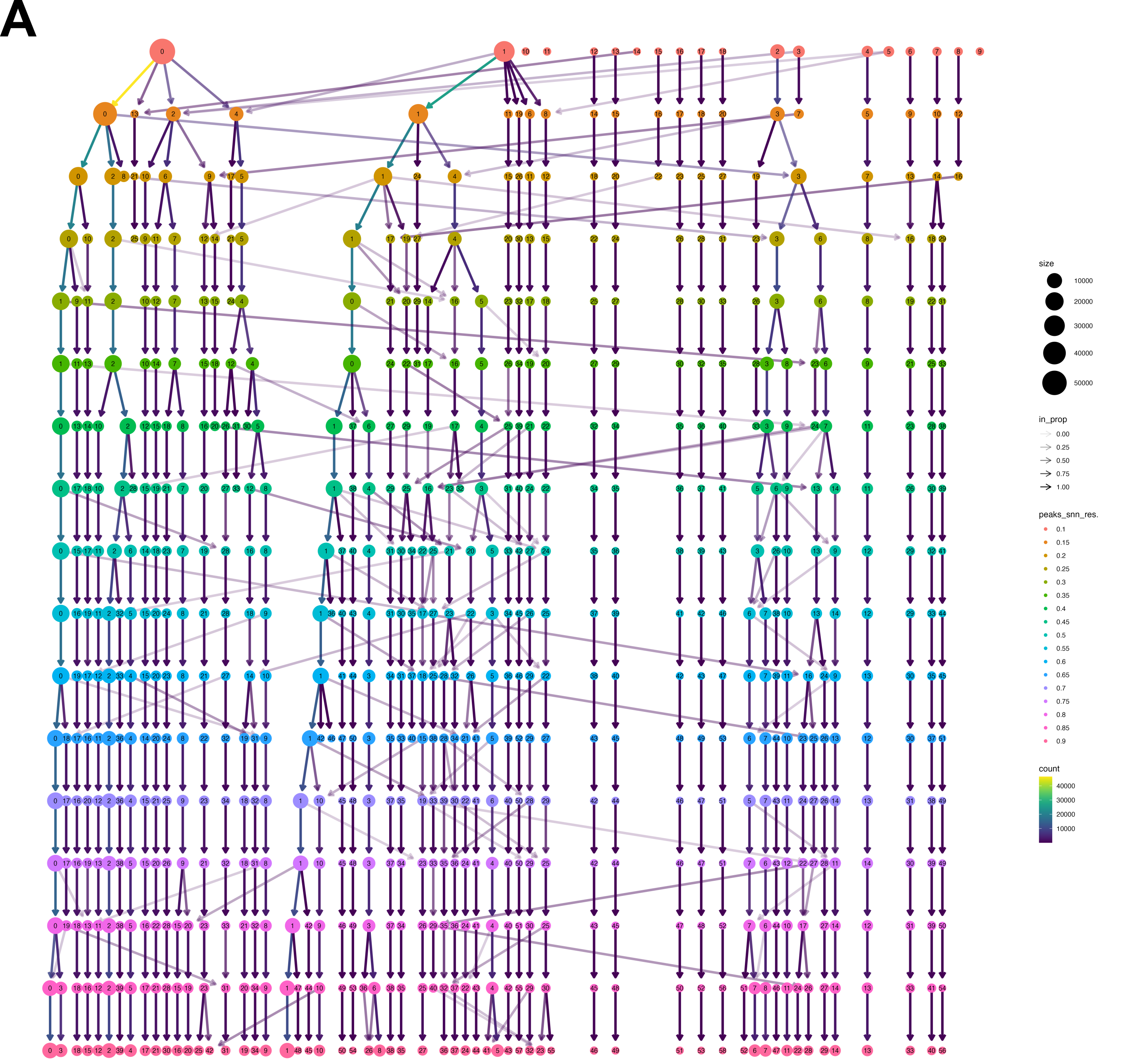

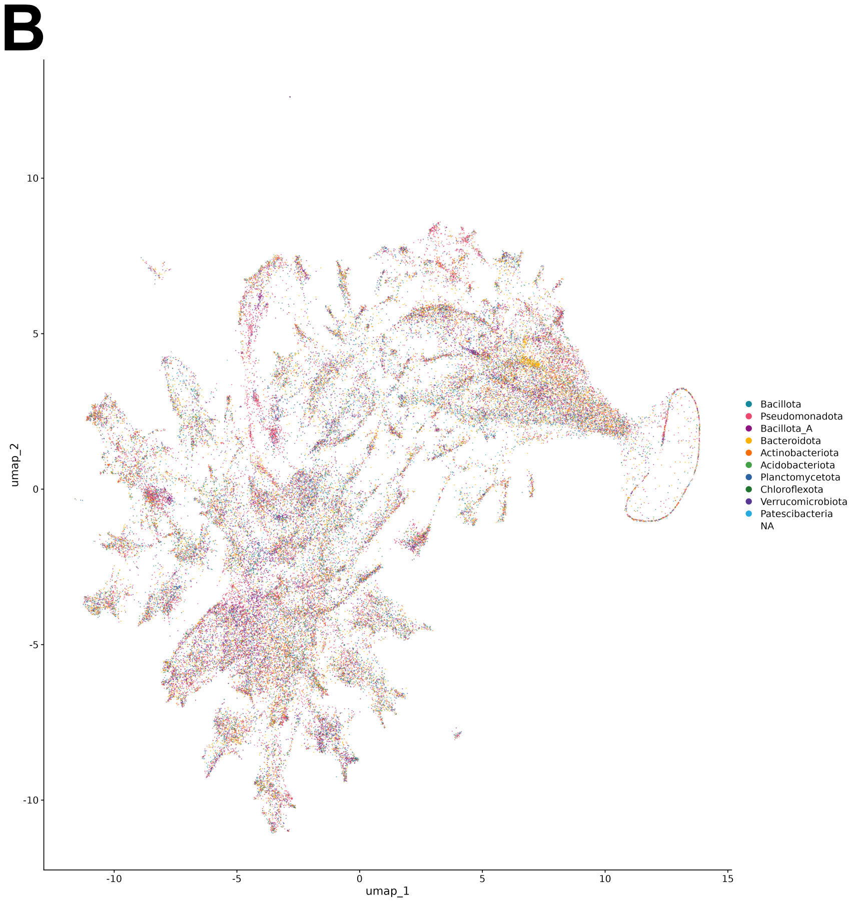

Supplementary Figure S7. (A) Clustering tree for (B) the Prophage-DB prophage UMAP, colored by phylum. For (B), only the top 10 phyla are shown (the rest are labeled as “NA”). For (A), “size” = the number of gene clusters in a particular cluster; “count” = the number of gene clusters carried over from a parent to child cluster; “in_prop” = the proportion of gene clusters carried over from a parent to a child cluster; “peaks_snn_res.” = the clustering resolution (as set in Seurat’s FindCluster() function).
