# Supplementary material for: Fast, flexible gene cluster family delineation with IGUA
Source: Nucleic Acids Res. 2026 Oct 13;54(19):gkag975. doi: 10.1093/nar/gkag975 (PMC13659240; doi:10.1093/nar/gkag975)
Supplement: Supplementary file 1 [file gkag975_supplemental_files.zip › Supplementary_Figure_S9_panel_datasets_v2.pdf]

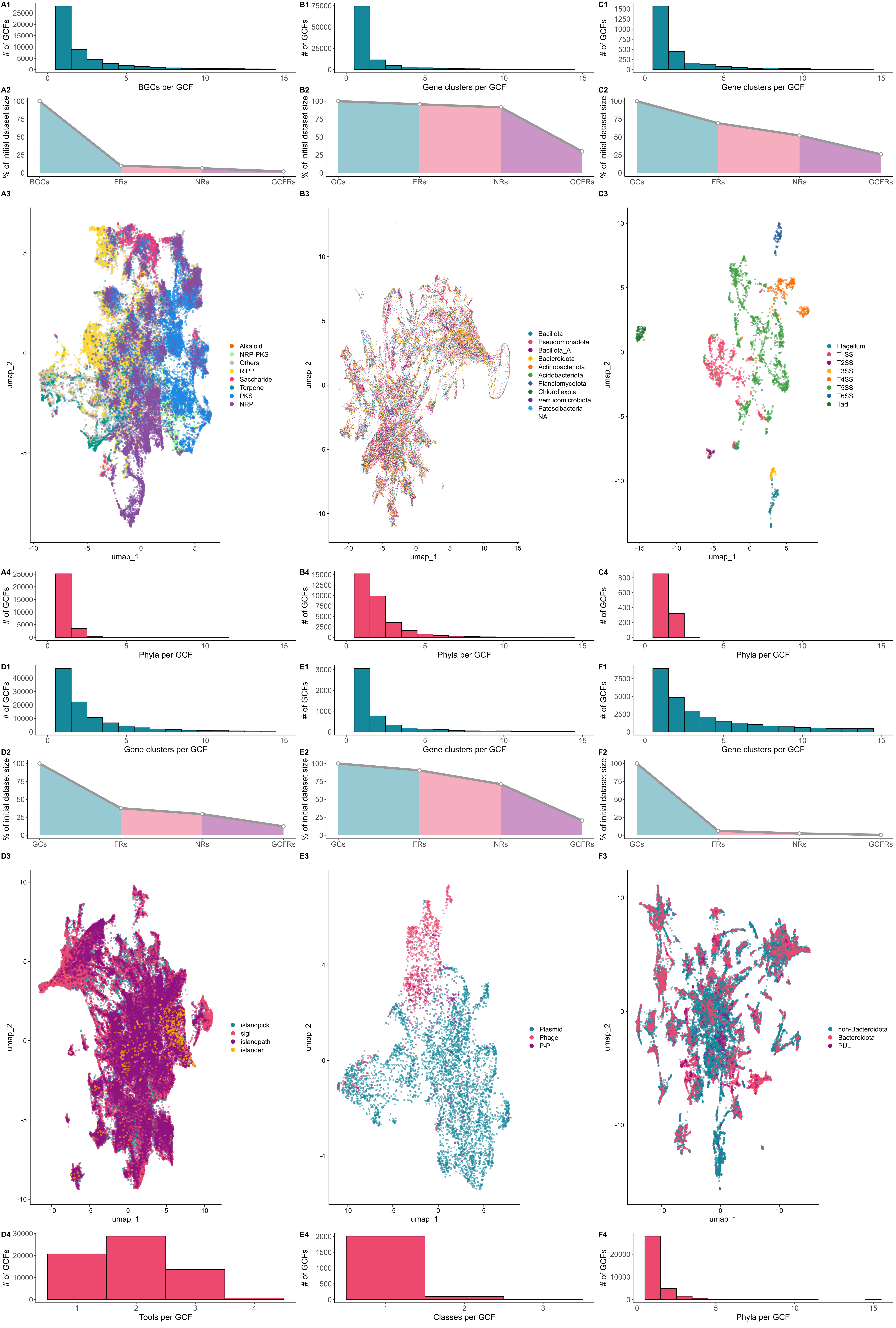

Supplementary Figure S9. IGUA was used to cluster (A) BGCs, (B) prophage, (C) single-locus secretion systems, (D) genomic islands, (E) phage-plasmids, and (F) CAZyme gene clusters into GCFs, with plots denoting the following: (1) histograms showing the number of BGCs/gene clusters (X-axes) per GCF (Y-axes); (2) area plots showing the reduction in data set size (% of initial data set size, Y-axes) at each step in IGUA's pipeline (X-axes). BGCs/gene clusters (GCs) correspond to the original input data set (i.e., 100% of the initial data set size), while FRs (fragment representatives), NRs (nucleotide representatives), and GCFRs (GCF representatives) correspond to the percentage of the input data set maintained at steps i (fragment removal), ii (nucleotide-level deduplication), and iii (protein-level clustering) in the IGUA pipeline, respectively; (3) BGC/gene cluster UMAPs of IGUA GCF representatives (points), colored by GCF majority class (A = biosynthetic class; B = phylum (only top 10 are shown); C = secretion system type; D = detection tool; E = gene cluster class; F = phylum (categorized into Bacteriodota, non-Bacteriodota and PUL gene clusters)); (4) histograms showing the number of phyla (A-C and F; X-axes), tools (D; X-axis), or class (E; X-axis) spanned by each GCF (Y-axes). For panels labeled 4, singletons have been excluded. For panels labelled 1 and 4, the X-axes have been limited to 15.
